# Rescaling the trophic structure of marine food webs

**DOI:** 10.1111/ele.12226

**Published:** 2013-12-06

**Authors:** Nigel E Hussey, M Aaron MacNeil, Bailey C McMeans, Jill A Olin, Sheldon FJ Dudley, Geremy Cliff, Sabine P Wintner, Sean T Fennessy, Aaron T Fisk

**Affiliations:** 1 Great Lakes Institute for Environmental Research, University of Windsor401 Sunset Avenue, Windsor, ON N9B 3P4, Canada; 2 Australian Institute of Marine SciencePMB No.3, Townsville MC, Townsville, QLD 4810, Australia; 3 Department of Integrative Biology, University of GuelphGuelph, ON N1G 2W1, Canada; 4 Department of Oceanography and Coastal Sciences, Louisiana State UniversityBaton Rouge, LA, 70803, USA; 5 Department of Agriculture, Forest and FisheriesPrivate Bag X2, Rogge Bay 8012, Cape Town, South Africa; 6 KwaZulu-Natal Sharks BoardPrivate Bag 2, Umhlanga Rocks 4320, KwaZulu-Natal, South Africa; 7 Biomedical Resource Unit, University of KwaZulu-NatalPrivate Bag X54001, Durban 4056, South Africa; 8 Oceanographic Research InstitutePO Box 10712, Marine Parade, Durban 4056, South Africa

**Keywords:** Discrimination, fish, food webs, marine, shark, stable isotopes, trophic level, trophic position

## Abstract

Measures of trophic position (TP) are critical for understanding food web interactions and human-mediated ecosystem disturbance. Nitrogen stable isotopes (δ^15^N) provide a powerful tool to estimate TP but are limited by a pragmatic assumption that isotope discrimination is constant (change in δ^15^N between predator and prey, Δ^15^N = 3.4‰), resulting in an additive framework that omits known Δ^15^N variation. Through meta-analysis, we determine narrowing discrimination from an empirical linear relationship between experimental Δ^15^N and δ^15^N values of prey consumed. The resulting scaled Δ^15^N framework estimated reliable TPs of zooplanktivores to tertiary piscivores congruent with known feeding relationships that radically alters the conventional structure of marine food webs. Apex predator TP estimates were markedly higher than currently assumed by whole-ecosystem models, indicating perceived food webs have been truncated and species-interactions over simplified. The scaled Δ^15^N framework will greatly improve the accuracy of trophic estimates widely used in ecosystem-based management.

## Introduction

Detecting and measuring food web dynamics is highly complex given heterogeneous environments, multiple functional groups and multi-level species interactions. The theory of trophic dynamics (Lindeman 1942) provided an important conceptual framework for understanding this complexity by conceiving the flow of energy through an ecosystem in the form of discrete trophic levels (TLs). This theory of energy transfer evolved into trophic position (TP), a quantitative, continuous (fractional) measure of the hierarchical role of a given species within a food web that, unlike TL, explicitly recognizes that species feed omnivorously (Vander Zanden & Rasmussen 1996). Food webs are accordingly viewed as a framework consisting of discrete TL consumers or known functional groups within which individual species are measured on a continuous scale of TP. The TP concept has enabled unique insights into the functioning of food webs including the length of food chains (Vander Zanden *et al*. 1999a), the role of bottom up or top down forcing in community structure (Menge & Sutherland 1976), levels of omnivory (Thompson *et al*. 2007) and the presence of trophic cascades (Bascompte *et al*. 2005). Importantly, TP has provided a standardized metric to investigate the effects of fishing (Pauly *et al*. 1998), altered trophic linkages (Vander Zanden *et al*. 1999b) and species removal (Myers *et al*. 2007), helping to shape modern conservation and ecosystem-based fisheries management (Pauly *et al*. 1998; Branch *et al*. 2010). Individual species TP and overall community trophic structure provide powerful means for monitoring human- and climate-mediated disturbance effects and the persistence and resilience of food webs (Rooney *et al*. 2008).

Conventionally, TP is calculated from stomach contents, whereby the proportional contributions of identified prey items are categorized into broad functional TP prey groups, providing an aggregate TP value for the consumer. This approach requires sacrificing large numbers of animals to characterize diet and is biased towards recent meals and the prey type consumed (i.e. hard or soft bodied). Moreover, stomach contents can generate suboptimal estimates of TP because diet items are typically organized into broad functional groups that do not reflect the true range of prey TPs (Hussey *et al*. 2011). To address these problems, naturally occurring nitrogen stable isotopes (δ^15^N) have become a routine method to estimate TP based on the premise that they integrate the spatial and temporal diet signature of an organism (Vander Zanden & Rasmussen 1996; Post 2002). Critical to the application of δ^15^N for estimating TP is the assumption that the increase in δ^15^N between predator and prey (termed the diet-tissue discrimination factor – Δ^15^N) is consistent and known. Given the δ^15^N value of a primary consumer, the TP of consumers within a food web can be estimated by dividing their net δ^15^N value by a given Δ^15^N value (Post 2002).

**Figure 1:**
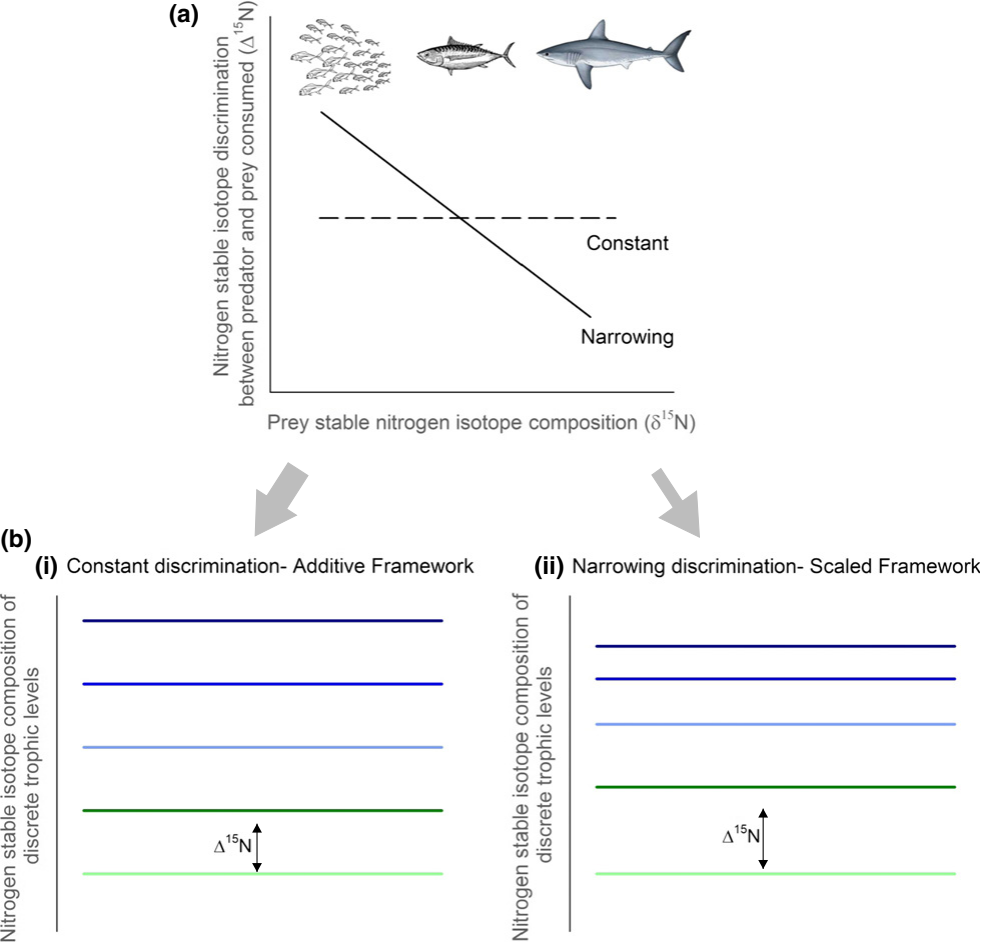
(a) Relationship concerning nitrogen stable isotope discrimination between predator and prey (Δ^15^N) and prey stable nitrogen isotope composition (δ^15^N). Constant discrimination assumes that enrichment in δ^15^N between predator and prey is independent of species and diet, whereas narrowing discrimination assumes that enrichment in δ^15^N between predator and prey is dependent on the inverse Δ^15^N-dietary δ^15^N relationship (Overmyer et al. 2008; Caut et al. 2009; Dennis et al. 2010). (b) The standard additive framework for examining food web structure following Post (2002), depicting trophic level (TL) estimates based on constant nitrogen stable isotope discrimination between predator and prey (i), and an alternative scaled framework accounting for the reported Δ^15^N-dietary δ^15^N relationship, depicting TL estimates based on narrowing nitrogen stable isotope discrimination between predator and prey (ii).

Most commonly, a fixed Δ^15^N value of 3.4‰ is used to estimate relative species TP and to reconstruct food web structure based on the pragmatic assumption of a Δ^15^N average across all components of the food web (Minagawa & Wada 1984; Post 2002; Fig. 1b(i)). Using a fixed Δ^15^N value at every TL, the assumed trophic structure of food webs is additive, with the same Δ^15^N value applied throughout the food web. Yet dedicated, controlled experimental studies have shown that Δ^15^N values can vary greatly among species and across taxa (Table S1), undermining the use of a fixed Δ^15^N value (Caut *et al*. 2009). Moreover, meta-analysis of experimental data (Robbins *et al*. 2005; Caut *et al*. 2009; Fig. 1a) and controlled experimental work (Caut *et al*. 2008; Overmyer *et al*. 2008; Dennis *et al*. 2010; Robbins *et al*. 2010) have shown a significant inverse relationship between Δ^15^N and the δ^15^N value of diet (herein referred to as the dietary δ^15^N value). Such variable ^15^N enrichment suggests discrimination is a dynamic, rather than a constant, equilibrium process (Olive *et al*. 2003). Consequently, ignoring variation in Δ^15^N values driven by the dietary δ^15^N value consumed may lead to major biases in the quantification of food web structure using a constant value of 3.4‰ per TL, underestimating TP of top predators and compressing food web length. By scaling TLs within isotope-derived food webs relative to dietary δ^15^N, Δ^15^N values will narrow with increasing TL, with notable effects throughout the food web (Fig. 1b(ii)).

Accurate trophic structure is important as marine ecosystem models commonly assume a four TL food web framework, from primary producers and herbivores (zooplankton) to primary and secondary consumers (e.g. teleost fishes, sharks, seabirds and mammals), with polar bears (*Ursus maritimus*) and killer whales (*Orcinus orca*) often the only fifth level consumers identified. The popular ECOSIM and ATLANTIS models typically apply this structure, with large carnivorous sharks feeding around TL four (Cortés 1999). Yet, the designation of large predatory sharks as largely secondary consumers directly contradicts known feeding behaviour (Wetherbee *et al*. 2012) and this kind of trophic aggregation can be a major source of poor ecosystem model performance (Fulton *et al*. 2003).

Here, we reconsider the currently perceived trophic structure of marine food webs through assessing whether a fixed ‘constant’ Δ^15^N value to quantify TP remains relevant given recent experimental advances in isotopic ecology. Through meta-analysis of Δ^15^N vs. dietary δ^15^N relationships we estimate consumer TP using an integrated δ^15^N-dependent enrichment model that allows for narrowing Δ^15^N values with increasing dietary δ^15^N. The resulting scaled Δ^15^N framework accounts for the downward bias and compression of TP among apex consumers, and the inconsistence of δ^15^N-based TP estimates dependent on the selected baseline organism. We compare TP estimates derived using the scaled Δ^15^N method for known low and high TP fishes (zooplanktivores and large predatory sharks) with TP estimates using the conventional constant Δ^15^N approach in two distinct marine ecosystems – subtropical KwaZulu-Natal in South Africa and Cumberland Sound in the Canadian Arctic.

## Methods

In summary, a range of consumers spanning zooplankton to apex predators were sampled in two distinct marine food webs and analysed for δ^15^N. Following a review of experimental data, a meta-analytical model of Δ^15^N vs. dietary δ^15^N values was integrated into a dietary δ^15^N value-dependent enrichment model based on an alternative form of the Von Bertalanffy growth curve to estimate TP based on the premise of continuous narrowing discrimination with increasing dietary δ^15^N values. These TP estimates were then compared to conventional TP estimates based on a constant discrimination value in an additive framework. The following methodological steps were undertaken.

### Sample collection

All zooplankton, teleost and elasmobranch species were sampled from the KwaZulu-Natal continental shelf food web in South Africa and from Cumberland Sound, Baffin Island in the Canadian Arctic. Zooplankton were sampled at both study sites via horizontal and vertical plankton tows. Teleosts were sampled from by-catch in the shallow water prawn trawl fishery, spear catches, and recreational fishermen/scientific catches in South Africa and via gill net, dip net, or bottom long-line sets in the Canadian Arctic (Table S2). For elasmobranchs, all South African species were sampled from captures in beach protection nets along the KwaZulu-Natal coast with the exception of whale sharks (*Rhincodon typus*) sampled from beach strandings. Canadian Arctic elasmobranchs, Arctic skate (*Amblyraja hyperborea*) and Greenland shark (*Somniosus microcephalus*), were sampled via bottom long-line. All teleosts and elasmobranchs were measured and a white muscle tissue sample was taken anterior to the first dorsal fin (or from the wing margin for rays/skates) and frozen (−20 °C).

All muscle tissue samples were lyophilized, homogenized and lipid extracted following standard chloroform–methanol practices and analysed for stable isotopes (δ^15^N and δ^13^C; see Supplementary Material file S3).

### Narrowing diet-tissue discrimination factor (Δ^15^N) meta-analytical model: Δ^15^N vs. dietary δ^15^N values

Using the ISI Web of Knowledge electronic database a standard literature search was conducted for all experimental work on Δ^15^N values in fish. Relevant terms used in the search included; δ^15^N, δ^13^C, stable nitrogen, stable carbon, diet-tissue discrimination factor, isotopic fractionation, and isotopic enrichment. All literature for experimentally derived Δ^15^N values for muscle tissue and whole fish of marine and freshwater origin fed on both natural and artificial diets were compiled (Table S1). Only data for whole fish/fish muscle tissue were retained, given known taxa and tissue specific Δ^15^N vs. dietary δ^15^N relationships (Caut *et al*. 2009). Using a series of hierarchical linear models the relationship between Δ^15^N vs. dietary δ^15^N values was estimated, along with potential effects of artificial vs. natural diets and marine vs. freshwater life history. Potential bias of studies that did not report reaching isotopic equilibrium was tested and did not alter our results.

For each experimental study, the uncertainty in observed diet and consumer δ^15^N (δ^15^N_d,i_ and δ^15^N_c,i_) was included as a study-level error that was assumed known (σ_d,i_ and σ_c,i_,) with the linear relationship between Δ^15^N and dietary δ^15^N estimated from latent states (θ_d,i_ and θ_c,i_) in a hierarchical model (Gelman *et al*. 2006). The meta-analytical model was then


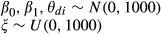



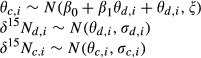


with intercept β_0_ and slope β_1_ characterizing the change in Δ^15^N as dietary δ^15^N values increase. Where present, additional β terms were included for covariates relating to diet source and life history.

### Scaled Δ^15^N framework based on a dietary δ^15^N value-dependent Δ^15^N model

As a negative linear Δ^15^N vs. dietary δ^15^N relationship implies a limit in δ^15^N values, we developed a dietary δ^15^N value-dependent enrichment model based on an alternative form of the von Bertalanffy growth equation:


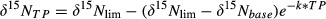


with δ^15^N_TP_ being the consumer isotope value at a given TP, δ^15^N_lim_ the saturating isotope limit as TP increases, δ^15^N_base_ the isotope value for a known baseline consumer in the food web, and *k* the rate at which δ^15^N_TP_ approaches δ^15^N_lim_ per TP step. This model is value-dependent in that δ^15^N_lim_ is reached when the rates of ^15^N and ^14^N uptake balance those of ^15^N and ^14^N elimination (i.e. Δ^15^N = 0 at δ^15^N_lim_), the rates of which are assumed constant among consumers and diets (see Supplementary Material S4).

Solved for TP this equation becomes


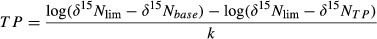


Calculating TP from this model requires estimates of both δ^15^N_lim_ and *k* which are given from the meta-analysis as


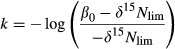



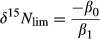


The estimated scaled TP (TP_scaled_) for each consumer is then estimable from the posterior distribution of the meta-analysis, given its δ^15^N value (δ^15^N_TP_) and a δ^15^N_base_ value for a given food web. Baseline TL2 consumers used to estimate δ^15^N_base_ were zooplankton, including copepod, *Euphausia frigida* and mysid, *Undinula vulgaris* for the South African food web and copepod, *Calanus hyperboreus* for the Canadian Arctic food web. The full meta-analytical model was implemented in a Bayesian framework, using the PyMC package (Patil *et al*. 2010) for the Python programming language. The model was run for 100,000 iterations, with an 80,000 iteration burn-in and thinned by a factor of 10; convergence was assessed through visual inspection of chains, plots of the model fit with the data, and Bayesian *P*-values (Gelman *et al*. 2006). Model code is included in Supplementary Materials S5.

### Testing the robustness of a scaled Δ^15^N framework

To test the utility of our scaled Δ^15^N framework, we compared TP_scaled_ values for species with uniquely well-characterized diets to those expected given their known feeding behaviour. Low TP species with well-characterized diets were zoopanktivores (TL3); in South Africa, African sardine (*Sardinops sagax*), whale shark and mobula rays (*Mobula* spp.), and in the Canadian Arctic, capelin (*Mallotus villosus*) and herring (*Clupea harengus*). High TP species were apex predators known to feed on a combination of elasmobranchs, marine mammals, and piscivorous teleosts, and therefore feed above TL4. These included white (*Carcharodon carcharias*), shortfin mako (*Isurus oxyrinchus*), bull (*Carcharhinus leucas*), pigeye (*Carcharhinus amboinensis*) and sand tiger (*Carcharias taurus*) sharks in South Africa and Greenland shark in the Canadian Arctic. In addition to the species with well-characterized diets, the fit of δ^15^N values for common prey of the high TP species were compared with consumers within the scaled Δ^15^N framework to assess individual/mean prey TP relative to individual/mean predator TP. Summarized stomach content data for all selected species are included in Table S6.

### Trophic position calculated with a scaled Δ^15^N framework vs. a standard additive Δ^15^N framework

To evaluate the difference between our scaled Δ^15^N framework and conventional TP estimates, the TP of individual fish was calculated according to Post (2002) using a constant Δ^15^N value of 3.4‰ (TP_additive_):


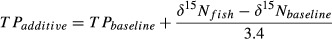


where δ^15^N _baseline_ and TL_baseline_ are the δ^15^N value and known TL for a low trophic level or baseline organism in the food web (e.g. zooplankton = TL2). TP estimated using both the scaled and standard additive Δ^15^N frameworks were then directly compared to examine variation between methods for both food webs.

Given that TP can be estimated from either a TL2 or TL3 baseline organism (Post 2002), TPs were calculated using both scaled and standard additive methods starting from TL2 (zooplankton – see above) and TL3 zooplanktivores (TL3; South Africa: whale shark, devil ray and South African sardine; Canadian Arctic: capelin and herring). The TL3 baseline-derived TPs were then compared to our TL2 baseline-derived estimates to determine if both the TL of the baseline organism and associated δ^15^N value and TP method affected TP estimation.

## Results

**Figure 2:**
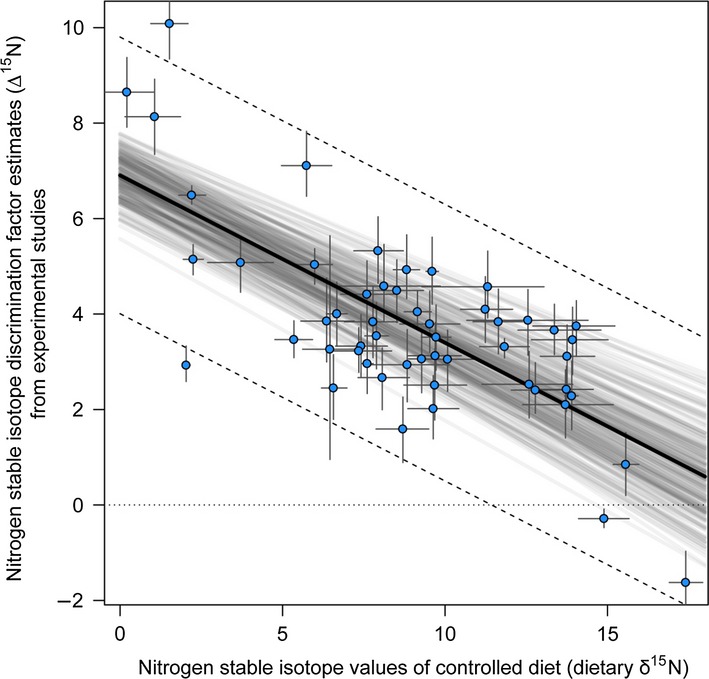
Posterior estimated relationship between nitrogen stable isotope discrimination factor estimates (Δ^15^N) from studies of marine and freshwater fishes fed known nitrogen stable isotope (δ^15^N) diets under controlled experimental conditions (see Table S1). As predicted by previous studies of Δ^15^N enrichment (i.e., Caut et al. 2009), a significant inverse relationship between the derived Δ^15^N and the δ^15^N values of diet for all experimental data was observed (see Results). Lines are posterior median values (thick line), 95% prediction intervals (dotted lines) and 95% uncertainty intervals (faded lines) given by 200 samples from the posterior distribution of the meta-analytical regression. Points are posterior medians and 95% uncertainty intervals for the latent state of diet and Δ^15^N in each study, assuming the study standard errors are known.

The meta-analytical model demonstrated that consumer discrimination is not constant but narrows with increasing dietary δ^15^N, a result of a negative linear function between Δ^15^N and dietary δ^15^N values (*n* = 59; Fig. 2). There was no substantive effect on the Δ^15^N vs. diet δ^15^N relationship relating to diet type (artificial vs. natural; ΔAIC = 4.4 over basic hierarchical model) or environment (marine vs. freshwater; ΔAIC = 11.2), consequently the final model used to calculate the Δ^15^N values within the scaled trophic framework included all experimental data (β_0_ = 5.92 [4.55, 7.33], β_1_ = −0.27 [−0.41, −0.14]; highest posterior density median [95% uncertainty intervals]).

Fifty-one species (34 teleosts and 17 elasmobranchs; *n* = 433) were sampled from South Africa along with seven species (five teleosts and two elasmobranchs; *n* = 193) from the Canadian Arctic. South African δ^15^N values in fish ranged from 9.5‰ for an individual whale shark to 17.3‰ for an individual white shark, with baseline zooplankton (TL2) at 5.2 ± 0.8‰ (mean ± 1 SD; *n* = 16). Canadian Arctic δ^15^N values ranged from 13.1‰ for capelin to 18.7‰ and 18.8‰ for Greenland shark and Greenland halibut (*Rheinhardtius hippoglossoides*), respectively, with baseline zooplankton at 10.2 ± 0.5‰ (*n* = 20).

**Figure 3:**
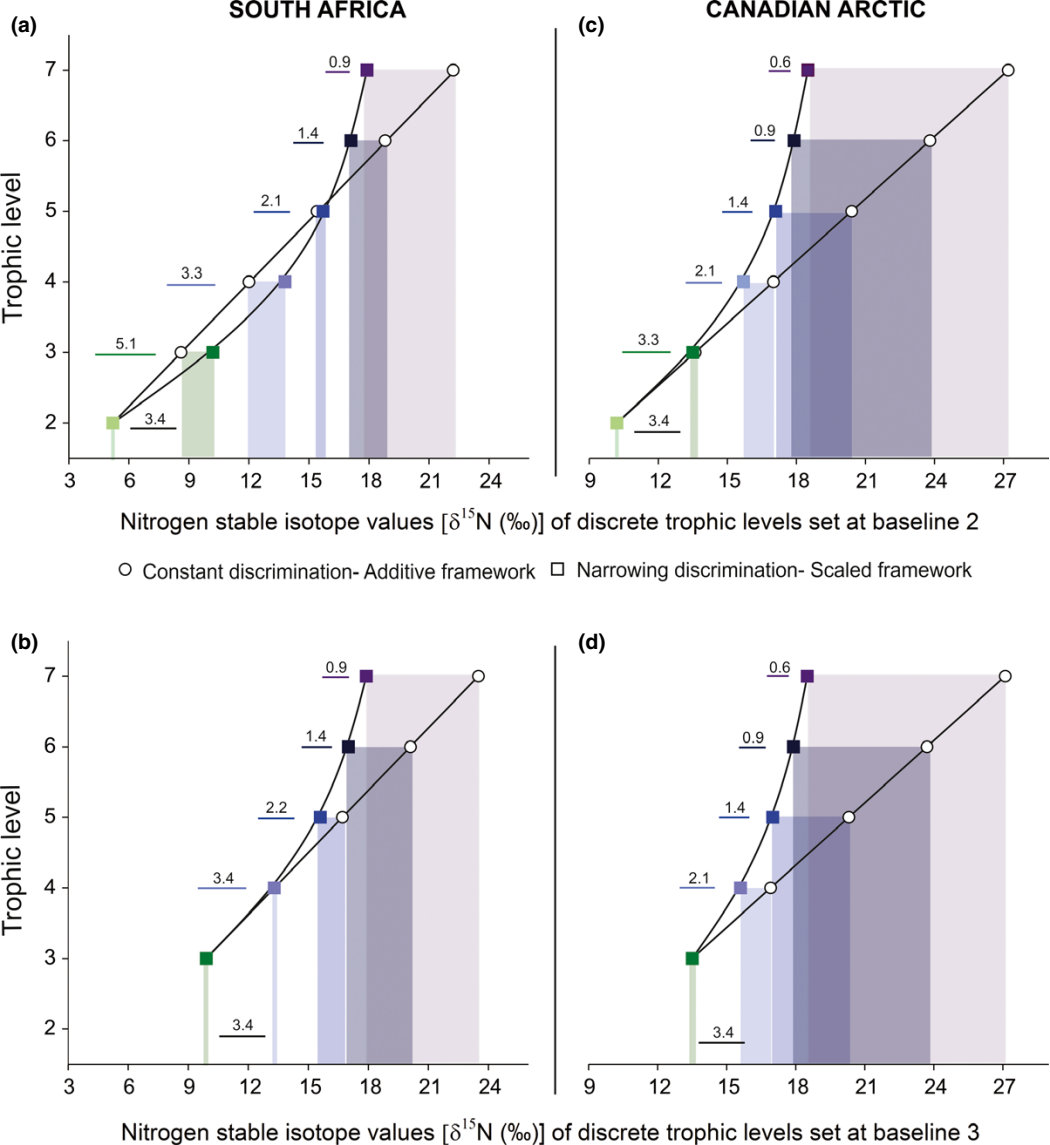
Relationship between trophic level and nitrogen stable isotope values of discrete trophic levels (TL) set from baseline 2 (a), (c) and 3 (b), (d) derived from constant discrimination-additive framework and narrowing discrimination-scaled framework for the South African and Canadian Arctic food webs. Shaded boxes show the difference in nitrogen stable isotope values of the TLs between methods for each baseline consumer. Numerical values represent the nitrogen stable isotope discrimination values calculated using the constant and narrowing discrimination methods among TLs.

For the scaled Δ^15^N framework, the estimated narrowing Δ^15^N values for discrete TL consumers in both food webs were markedly different to the assumed constant Δ^15^N value of 3.4‰. Starting from a TL2 baseline consumer (zooplankton) in South Africa, the scaled Δ^15^N framework estimated Δ^15^N values ranging from 5.1‰ for a discrete TL3 consumer to 0.9‰ for a discrete TL7 consumer, and 3.3‰ (TL3) to 0.6‰ (TL7) in the Canadian Arctic (Fig. 3a,c). Variation in discrete TL consumer narrowing Δ^15^N values between ecosystems was driven by system-specific baseline zooplankton δ^15^N values. Starting from a TL3 baseline consumer (zooplanktivores), narrowing Δ^15^N values within the scaled Δ^15^N framework were nearly identical in both ecosystems to those estimated from a TL2 baseline resulting in equivalent discrete TL consumer δ^15^N values (TL4-7; Fig. 3). In contrast, a fixed Δ^15^N value of 3.4‰ within the additive Δ^15^N framework generated variable δ^15^N values for discrete TL consumers that were dependent on the starting baseline (TL2 or TL3; Fig. 3a,b). For example, in the South Africa food web TL6 consumer δ^15^N values were 18.8‰ and 20.1‰ using the additive framework and baseline of TL2 and TL3, respectively; a discrepancy not observed within the scaled Δ^15^N framework (Fig. 3a,b).

**Figure 4:**
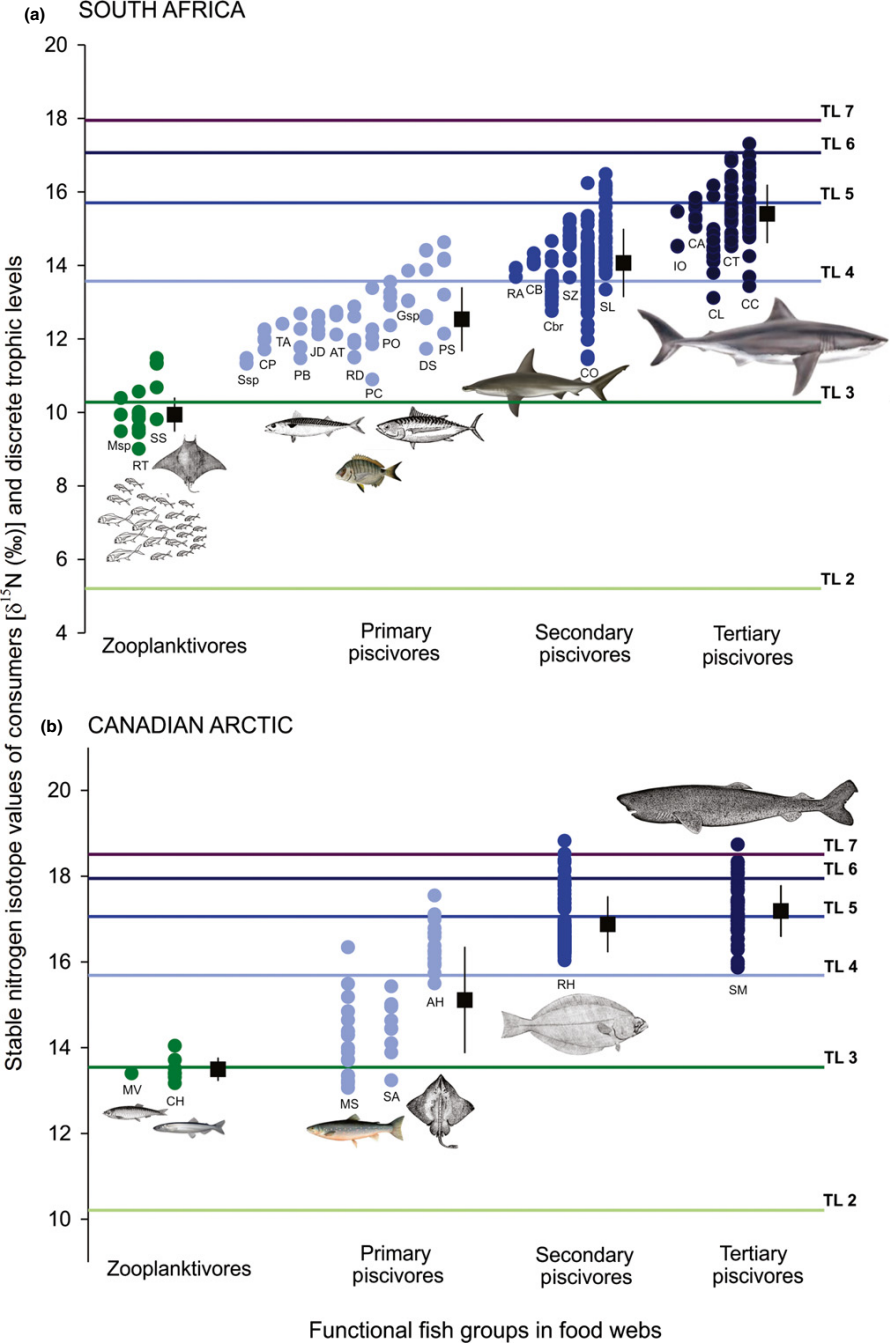
The structure of the (a) South African and the (b) Canadian Arctic marine food webs, depicting a scaled framework for estimating trophic position (TP), based on narrowing discrimination among trophic levels (TLs). Each dot represents trophic position estimates of individual fish per species characterized into four functional fish groups (color-coded solid points) and the functional fish group mean (± SD; filled squares) based on diet (see Table S6 and are calculated from the δ^15^N values of a baseline 2 consumer (zooplankton) at each respective location (TL = 2). Species included in the figure (left to right for each functional group) are listed as follows (see Table S1 for common names): (A) South African food web: Zooplanktivores— *Mobula* sp.; *Rhincodon typus*; *Sardinops sagax*; Primary piscivores— *Sarpa salpa*; *Chrysoblephus puniceus*; *Thunnus albacares*; *Pteromylaeus bovinus*; *Johnius dorsalis*; *Argyrosomus thorpei*; *Rhynchobatus djiddensis*; *Pomadasys commersonni*; *Pomadasys olivaceus*; *Galeichthys* sp.; *Diplodus sargus*; *Pomadasys striatum*; Secondary piscivores—*Rhizoprionodon acutus*; *Carcharhinus brachyurus*; *Carcharhinus brevipinna*; *Sphyrna zygaena*; *Carcharhinus obscurus*; *Sphyrna lewini*; Tertiary piscivores—*Isurus oxyrinchus*; *Carcharhinus amboinensis*; *Carcharhinus leucas*; *Carcharias taurus*; *Carcharodon carcharias*. (B) Canadian Arctic food web: Zooplanktivores— *Mallotus villosus*; *Clupea harengus*; Primary piscivores—*Myoxocephalus scorpius*; *Salvelinus alpinus*; *Amblyraja hyperborea*; Secondary piscivore—*Reinhardtius hippoglossoides*; Tertiary piscivore— *Somniosus microcephalus*.

**Figure 5:**
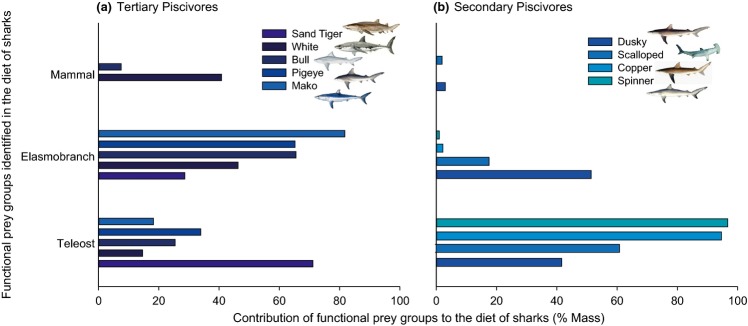
Mass contribution (%) of functional prey groups to the diet of (a) tertiary piscivores and (b) secondary piscivores sampled from the South African food web, based on stomach content analyses (see corresponding references in Table S6) illustrating how the dietary differences among these consumer groups influence trophic level estimates.

The TP estimates of species with well-characterized diets were more accurately described by the scaled Δ^15^N framework compared to the additive Δ^15^N framework in both South Africa and the Canadian Arctic and were in agreement with known diets (Fig. 4a,b; Table S6). For the scaled Δ^15^N framework, TP values of known zooplanktivores (TL3) in both systems ranged from 2.8 to 3.3 (Fig. 4a,b) even though the narrowing TL3 Δ^15^N value differed between systems (5.1‰ in South Africa vs. 3.3‰ in Canadian Arctic). Among apex predators (TL > 4), TP ranges were 4.0–7.7 for shortfin mako, pigeye, bull, sand tiger and white sharks in South Africa and for Greenland sharks in the Canadian Arctic, and showed large intraspecies variation (Fig. 4a,b). The mean TP of common secondary piscivore prey in South Africa, including scalloped hammerhead and dusky sharks, were 0.7TL below that of the mean apex predatory shark TP (Fig. 4a), in agreement with known percentage mass contribution of elasmobranchs, marine mammals, and teleosts in their diets (Fig. 5; Table S6). As expected, mean TP estimates of primary piscivores (mainly teleosts but including batoids) were approximately one TL below that of the secondary piscivores. In the Canadian Arctic, the hierarchical structuring of the food web using the scaled Δ^15^N framework reflected the diverse diets of teleost fishes in this depauperate system, but was consistent with ordered primary, secondary and tertiary piscivores and expected levels of omnivory (Fig. 4b).

**Figure 6:**
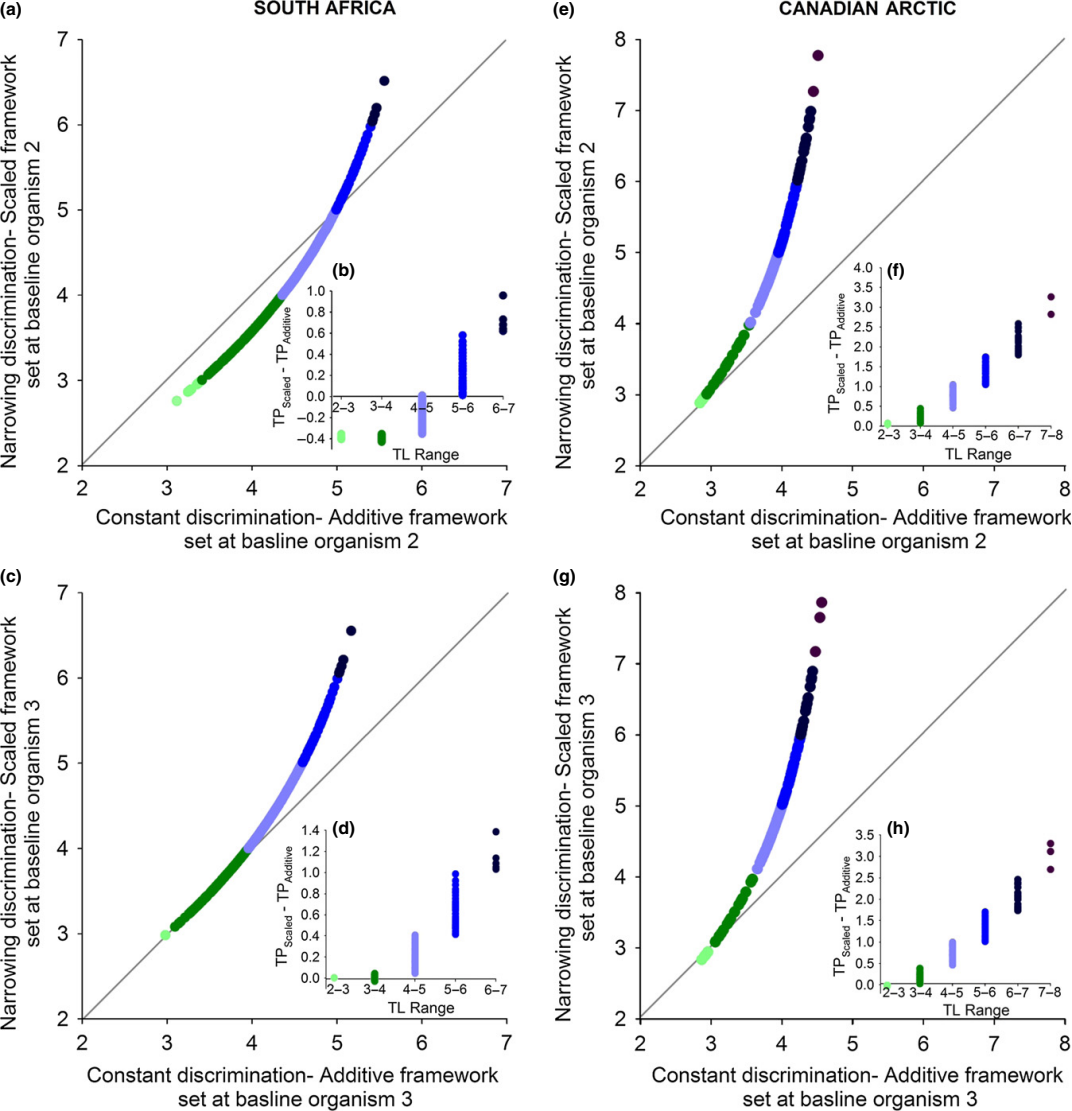
: Comparisons of fish trophic position (TP) estimates resulting from the constant discrimination-additive framework and the narrowing discrimination-scaled framework for the South African and Canadian Arctic food webs starting from baseline 2 (a), (c) and baseline 3 (b), (d) consumers and the corresponding differences in absolute values between methods for each baseline consumer (i-iv). The solid gray line represents the 1:1 relationship between the two TP estimation methods. Relationships are colored coded based on trophic level (TL) groups as follows: TL2 – light green; TL3 – dark green; TL4 – light blue; TL5 – royal blue; TL6 – dark blue; TL – 7 purple.

Compared to the scaled framework, the additive Δ^15^N framework overestimated the TP of lower TP organisms in South Africa by ∼ 0.4TL when starting from a TL2 baseline (Fig. 6a, b), while from a TL3 baseline, upper predator TPs were underestimated by an average of ∼ 0.6TL and for an individual animal up to 1.4TL (Fig. 6c, d). For the Canadian Arctic, species > TL3 were underestimated from both baselines using the additive Δ^15^N framework compared with the scaled Δ^15^N framework, with the difference in TP increasing exponentially up to a maximal error of 3.5TL for an individual fish (Fig. 6e–h).

## Discussion

Trophic dynamic theory underpins understanding of food web structure and species interactions that ultimately shape modern marine ecosystem ecology, conservation and management (Treblico *et al*. 2013). Our data determine that the commonly perceived trophic structure of marine food webs is truncated and species interactions, measured on a continuum from lower to higher feeding nodes, oversimplified. The scaled Δ^15^N framework, based on narrowing discrimination and presented in two distinct marine ecosystems, introduces a powerful and widely applicable approach to quantify trophic position and structure. The restructuring of food webs is upheld by several lines of evidence including (1) viable representation of high TP fish (> TL4) predator–prey feeding relationships that concur with known feeding behaviour; (2) accurate estimation of zooplanktivores (TL3) despite independently scaled Δ^15^N frameworks across ecosystems; (3) accurate representation of species ontogenetic and omnivorous variation; and (4) insensitivity of the scaled framework to the starting baseline used (TL2 or TL3) and the respective δ^15^N value of the baseline organism.

The scaled Δ^15^N framework TP estimates of apex predatory sharks were markedly higher than those derived from either the standard additive Δ^15^N framework or conventional stomach content TP estimates, and are validated by known predator–prey relationships (Figs 4 and 5; Table S6). The diet of apex predators in South Africa is dominated by elasmobranchs (29–80% mass; Fig. 5; Table S6) and equates well with the mean scaled TP of apex sharks (5.1 ± 0.5) and common shark prey species (4.4 ± 0.4) given known levels of omnivory (Table S6). These TP estimates conflict with conventional stomach content TP estimates of 4.4 ± 0.1 and 4.2 ± 0.1 for predators and prey, respectively (Cortés 1999), which suggest feeding over an unrealistic range of 0.2TL. Moreover if large sharks indeed feed between TL 4.1–4.5 their diet would consist predominantly of small zooplanktivorous fish (TL3), directly contradicting detailed dietary studies. Such results help explain erroneous TP estimates of 4.0 and 4.3 for Greenland shark, using surrogate additive Δ^15^N values of 3.8‰ and 4.0‰, that have been strongly questioned for failing to equate with known predation on marine mammals (Fisk *et al*. 2002; McMeans *et al*. 2010; MacNeil *et al*. 2012; Table S3). Similarly, in the northeast Atlantic, TP of shortfin mako, was estimated at 4.0 using a Δ^15^N of 3.4‰ (Estrada *et al*. 2003), placing them one TL above zooplanktivores, which is inconsistent with their known diet of piscivorous bluefish (*Pomatomus saltatrix*; Table S6).

Equally, the scaled Δ^15^N framework estimates of zooplanktivore TP were accurately represented from a TL2 baseline despite the two study systems having markedly different δ^15^N baseline and associated narrowing discrimination values. In contrast, the additive framework δ^15^N value of a discrete TL2 consumer in South Africa back calculated from zooplanktivores (i.e. TL3) was 6.4‰, 1.2‰ higher than the observed mean zooplankton δ^15^N value of 5.2‰. This resulted in inaccurate characterization of food web structure from a TL2 baseline through overestimating lower TP fish and underestimating higher TP fish. Mancinelli *et al*. (2013) reported a similar inconsistency in a comparison of δ^15^N TP estimates of marine fish from base consumers (TL1) and primary consumers (TL2), related to guild-specific fractionation between predator and prey.

When considering known feeding behaviours, intraspecies TP variability was well described by the scaled framework (see Table S2 for size ranges). Variation in TP can be driven by ontogenetic variation in feeding (Scharf *et al*. 2000) and generalist or specialist feeding behaviour within generalist populations (Araújo *et al*. 2011). Ontogenetic isotope profiles of shark and fish species (including the scalloped hammerhead, dusky and white shark in this study) have been related to diet shifts with size (Hussey *et al*. 2011, 2012a) and were accurately represented by the scaled approach. For Greenland shark, that feed on a broad prey base from zooplanktivores to marine mammals (Table S6; MacNeil *et al*. 2012), variable oxychlordane concentrations have suggested intraspecies dietary specialization on marine mammals that is unresolved by conventional isotope analysis (Fisk *et al*. 2002). Scaled Greenland shark TP estimates of 4.2–7.7 are consistent with these contaminant-based results. Moreover, large Greenland halibut (> 70 cm) are cannibalistic piscivores that also feed on fish offal and seal remains (Rodríguez-Marín *et al*. 1995; Jeremiah Young, pers. obs.). The scaled Δ^15^N framework accurately depicted Greenland halibut TP variation, a result of feeding on a diverse prey base in the sparse Arctic environment.

The choice of starting baseline (i.e., TL2 or 3) and associated δ^15^N value resulted in method-specific differences in TP estimation highlighting a discrepancy when using a constant δ^15^N value of 3.4‰. Assuming an additive Δ^15^N framework, there is no requirement to start from a designated point within the food web; TP can be calculated from either baseline TL2 or TL3 (Post 2002). Yet in South Africa, the δ^15^N values of the selected baseline organisms (5.2‰ – TL2 and 9.9‰ – TL3) resulted in inconsistent additive framework TLs throughout the food web. These inconsistencies resulted in consumer TP underestimation from baseline TL3 relative to TL2 when compared to the scaled framework, with the error increasing higher in the food web. For the Canadian Arctic, the additive and scaled Δ^15^N frameworks performed similarly for TL3 consumers. Moving progressively higher in the Arctic food web, narrowing Δ^15^N values were considerably lower than 3.4‰, a result of the high baseline δ^15^N value, leading to the additive Δ^15^N framework underestimating TP from both baselines. The scaled framework accounted for this baseline bias, with consistent narrowing Δ^15^N values and consumer TLs, starting from baseline TL2 and TL3 at both study sites.

An inherent problem with assigning discrete baseline organisms to estimate isotope based TP is the potential for temporal and spatial basal variation (Vander Zanden & Rasmussen 1999). When examining TP of apex predators such as sharks, residency and movement in multiple ecosystems may cloud assignment to one food web, a known caveat of isotopic food web analysis (Post 2002). While our scaled Δ^15^N framework accounts for TP error associated with the starting baseline used (i.e. TL2 or TL3), a measure of error associated with predators proportionally feeding in inshore and offshore food webs during the isotopic incorporation period may impact absolute TP values. Given most species studied spend the majority of their time in the continental shelf food web this TP error is negligible. For the Arctic food web, two predominantly benthic species were included, Greenland halibut and Arctic skate. Greenland halibut TP values were highly variable as would be expected given their broad diet (see above), but maximal TP may have been overestimated because of the pelagic baseline organism used. Nevertheless, the scaled framework Greenland halibut TP estimates were more realistic relative to known diet when compared to the additive approach. Incorporating multiple baseline organisms in to our scaled δ^15^N model to advance the constant discrimination dual baseline model proposed by Post (2002) will further refine TP estimation. Logistics of field sampling and complexities of defining appropriate baseline sources have commonly precluded the use of this approach. We suggest that through restructuring food webs, previously muted species interactions will be more readily identifiable enabling greater precision to determine discrepancies related to basal sources.

### Mechanism driving a scaled Δ^15^N value

Despite being underutilized, the Δ^15^N vs. dietary δ^15^N relationship that underpins our scaled Δ^15^N framework has been repeatedly observed (Caut *et al*. 2009; Robbins *et al*. 2005; Table S1). Similarly, compound-specific stable isotope analysis of individual amino acids (AA-CSIA) has found that the Δ^15^N values of predators fed high δ^15^N diets under controlled conditions were lower than the assumed constant value of 7.6‰ derived for low TP organisms (Germain *et al*. 2013), consistent with the trend seen in bulk isotope analyses. Despite mounting empirical support for this relationship, the mechanism behind the Δ^15^N vs. dietary δ^15^N relationship remains largely unknown. A validated mechanism for the broad application of a single, additive Δ^15^N value in ecology has also yet to be empirically shown, but rather is based on a pragmatic assumption (See further discussion in Supplementary Material S4 and S7).

### Implications of a rescaled food web

Theoretical models of food web structure use binary, flow-based or mass balance approaches that are generally based on stomach contents (Carscallen *et al*. 2012). These models are widely accepted decision-making tools used by resource managers to explore management scenarios that ultimately guide policy decisions (Fulton *et al*. 2011). Despite the intuitive understanding that sharks are apex predators in marine ecosystems, conventional parameterization of whole ecosystem models consistently place sharks and other large carnivorous teleosts within a narrow range of mid TP values (∼ TP 4). Without accurate characterization of TP among consumers, the food web component of whole ecosystem models will ultimately fail to provide reliable inferences for management decisions (Fulton *et al*. 2003, 2011).

Trophic position underestimation in ECOPATH is thought to result from the arbitrary assignment of baseline TL species such as detritus (at TL1) and the assumption of non-selective feeding on identified prey (Dame & Christian 2008). For standard stomach content calculated TP, coarse resolution grouping of prey items routinely truncates maximum TP estimates. For example, Cortés (1999) estimated the TP of sharks using a broad elasmobranch prey group (sharks and batoids; TP 3.6), rather than accounting for known species-specific feeding behaviours; i.e. tertiary vs. secondary piscivore sharks. For binary approaches, TP is calculated as the average of the shortest TP for the species and the prey-averaged TP, resulting in a bias towards shorter chain lengths that limits upper TP estimates (Williams & Martinez 2004; Carscallen *et al*. 2012). These methods of examining food web interactions naturally truncate food web length, producing averaged TP estimates that do not reflect the true TP range of individual species and the system as a whole.

In a conventional four TL ecosystem (Cox *et al*. 2002; Kitchell *et al*. 2002), models examining the effect of removing high TP fish on food web interactions have commonly not detected strong top down effects (Manickchand-Heileman *et al*. 1988; Kitchell *et al*. 2002), contrary to expectations (Stevens *et al*. 2000). However in an alternate model incorporating intraguild predation at TL4 (i.e. sharks eating sharks), strong nonlinear responses in the food web occurred (Kitchell *et al*. 2002). Through incorporating intraguild predation, Kitchell *et al*. (2002) ultimately expanded the trophic structure of the food web, an expansion consistent with the TP error estimates between the scaled and additive Δ^15^N frameworks for higher TP species. Underestimation of large fish TP also helps explain inverted biomass pyramids and the potential overestimation of upper predator fish biomass in remote marine ecosystems, leading to problems in measuring food chain length, energy flow pathways and levels of omnivory (Vander Zanden *et al*. 1999a; Treblico *et al*. 2013). The widespread truncation of marine food webs, which masks higher TLs and associated interactions, has important consequences not only for whole ecosystem models (Fulton *et al*. 2011), but also for monitoring the effects of global fisheries (Pauly *et al*. 1998; Branch *et al*. 2010); understanding the impact of invasive species; and modeling contaminant biomagnification (Burkhard *et al*. 2013). Moreover, elevated TPs of large apex predators indicates 1 to 2 orders of magnitude less energy available to support these fish than previously thought, assuming a transfer efficiency of approximately 10% (Treblico *et al*. 2013). This suggests that population numbers or biomass estimates of highly exploited large predators could be much smaller than current theoretical estimates, underscoring the need for proactive precautionary fisheries management to ensure viable populations.

Our restructuring of marine food webs using a scaled δ^15^N framework was based on fish given their biomass and diversity dominates consumer TLs in aquatic systems and their associated importance in structuring ecosystem processes. The presented framework to estimate TP is universally applicable to a broad range of marine taxa, including birds and marine mammals across aquatic environments. Meta-analysis of experimental data has shown that the Δ^15^N vs. dietary δ^15^N value is ubiquitous across taxa, but identifies taxa-specific regression coefficients (Caut *et al*. 2009). Integrating these taxa-specific coefficients into our dietary δ^15^N value-dependent enrichment model will enable TP estimation of the whole marine species complement within an ecosystem.

## Summary

Accounting for narrowing discrimination with increasing TL using a scaled Δ^15^N trophic framework provides an accurate representation of species structuring in aquatic ecosystems, resulting in markedly higher TP estimates for large fish and extending food web length compared to the conventional constant discrimination approach. This builds on previously scaled discrimination approaches, based on guild- and species-specific Δ^15^N values that better characterize food web interactions (Hobson *et al*. 1995; Madigan *et al*. 2012). By accounting for directional Δ^15^N narrowing within food webs, our ability to accurately measure absolute TP variation is substantially improved enabling ecologists and resource managers to better understand and conserve aquatic ecosystems.
