# Supplementary material for: Rescaling the trophic structure of marine food webs
Source: Ecol Lett. 2013 Dec 6;17(2):239–50. doi: 10.1111/ele.12226 (PMC3912912; doi:10.1111/ele.12226)
Supplement: Supplementary file 1 [file ele0017-0239-sd1.docx]

**Supplementary Material Table S1:** Data from experimental stable isotope feeding studies examining nitrogen stable isotope (δ^15^N) diet-tissue discrimination factors (Δ^15^N) in fish and included in the meta-analytical model to define the Δ^15^N vs. dietary δ^15^N relationship that incorporates continuous narrowing fractionation into a scaled Δ^15^N trophic framework (see Figure 2). Exp. – experimental conditions (controlled [C], semi-controlled [SC]; Equ. – reached equilibrium (equilibrium [E], unknown [U]), Env. – environment (freshwater [FW], marine [M]), Tis. – tissue (muscle [M], whole fish [W]), Lipid – lipid extraction (yes [Y]/ no [N]), Acid – acidification (yes [Y]/ no [N]), Precis. – analytical precision

| **CONSUMER** | | | | | | | | | | **PREY (DIET)** | | | | | |  | **DTDF** | | **REFERENCE** |
| --- | --- | --- | --- | --- | --- | --- | --- | --- | --- | --- | --- | --- | --- | --- | --- | --- | --- | --- | --- |
| **Common name** | **Scientific name** | **Exp.** | **Equ.** | **Env.** | **Tis.** | **Lipid** | **Acid** | **δ^15^N** | **δ^15^N**  **(SD)** | **Type** | **Diet** | **Lipid** | **Acid** | **δ^15^N** | **δ^15^N**  **(SD)** | **Precis.** | **∆15N** | **∆15N**  **(SD)** | **Study** |
| Cui-ui | *Chasmistes cujus* | SC | U | FW | M | N | N | 10.7 | 0.35 | Hatchery feed | F | N | N | 7.5 |  | 0.20 | 3.2 |  | Estep & Vigg 1985 |
| Cui-ui | *Chasmistes cujus* | SC | U | FW | M | N | N | 12.05 | 0.39 |  | F | N | N | 7.5 |  | 0.20 | 4.6 |  | Estep & Vigg 1985 |
| Cutthroat trout | *Oncorhynchus clarkii* | SC | U | FW | M | N | N | 7.93 | 1.58 |  | F | N | N | 6.5 |  | 0.20 | 1.43 |  | Estep & Vigg 1985 |
| Broad whitefish | *Coregonus nasus* | C | E | FW | M | N | N | 11.6 | 0.40 | Commercial trout food | F | N | N | 7.8 |  | 0.30 | 3.8 |  | Hesslein *et al.* 1993 |
| Broad whitefish | *Coregonus nasus* | C | E | FW | M | N | N | 12.7 |  | Lake trout | T | N | N | 9.7 |  | 0.30 | 3.1 |  | Hesslein *et al.* 1993 |
| Rainbow trout | *Oncorhynchus mykiss* | C | E | FW | M |  |  | 11.84 |  | Granular food | F |  |  | 9.30 | 0.20 | 0.12 | 2.97 |  | Pinnegar & Polunin 1999 |
| Rainbow trout | *Oncorhynchus mykiss* | C | E | FW | W |  |  | 11.4 |  | Granular food | F |  |  | 8.90 | 0.70 | 0.12 | 2.75 |  | Pinnegar & Polunin 1999 |
| Lake trout | *Salvelinus namaycush* | C | E | FW | M | C | N | 10.23 | 0.47 | Mixture |  | C | N | 6.41 | 0.42 | 0.20 | 3.82 |  | Harvey *et al.* 2002 |
| Lake trout | *Salvelinus namaycush* | C | E | FW | M | C | N | 13.14 |  | Mixture |  | C | N | 9.51 | 0.47 | 0.20 | 3.89 |  | Harvey *et al.* 2002 |
| Catfish | *Pterygoplichthys disjunctivus* | C | E | FW | M | Y | N | 7.39 | 0.16 | Algae | T | Y | N | 2.26 | 0.16 | 0.11 | 5.13 |  | German & Miles 2010 |
| Pumpkinseed | *Lepomis gibbosus* | C | U | FW | M | Y | N | 8.80 | 0.20 |  | T | C | N | 5.40 | 0.30 | 0.30 | 3.40 |  | Colborne & Robinson 2013 |
| Pumpkinseed | *Lepomis gibbosus* | C | U | FW | M | Y | N | 8.80 | 0.20 |  | T | C | Y | 2.20 | 0.20 | 0.30 | 6.50 |  | Colborne & Robinson 2013 |
| Rainbow trout | *Oncorhynchus mykiss* | C | E | FW | M | N | N | 11.4 |  |  | F | N | N | 7.90 | 0.20 | 0.20 | 3.5 |  | Heady & Moore 2013 |
| Rainbow trout | *Oncorhynchus mykiss* | C | E | FW | M | N | N | 16.20 |  |  | F | N | N | 13.90 | 0.10 | 0.20 | 3.4 |  | Heady & Moore 2013 |
| Catfish | *Clarias gariepinus* | C | E | FW | W | Y | N | 13.14 |  |  | F | Y | N | 10.14 |  |  | 3 | 0.06 | Enyidi *et al.* 2013 |
| Catfish | *Clarias gariepinus* | C | U | FW | W | Y | N | 9.00 |  |  | F | Y | N | 0.08 |  |  | 8.92 |  | Enyidi *et al.* 2013 |
| Catfish | *Clarias gariepinus* | C | U | FW | W | Y | N | 13.00 |  |  | F | Y | N | 5.62 |  |  | 7.38 |  | Enyidi *et al.* 2013 |
| Brook trout | *Salvelinus fontinalis* | C | U | FW | U | U | U | 13.05 |  |  | U | U | U | 8.50 |  |  | 4.55 |  | Peterson & Howarth 1987 |
| Eurasian perch | *Perca fluvia* | C | U | FW | M | Y | N | 10.49 |  |  | F | Y | N | 7.61 | 0.10 | 0.40 | 2.88 | 0.42 | Vollaire *et al.* 2007 |
| Grass carp | *Ctenopharyngodon idellus* | C | U | FW | M | N | N | 4.9 | 0.2 |  | F | N | N | 2.04 | 0.06 | 0.10 | 2.86 |  | Xia *et al.* 2013 |
| Grass carp | *Ctenopharyngodon idellus* | C | U | FW | M | N | N | 10.63 |  |  | F | N | N | 6.68 | 0.17 | 0.10 | 3.95 |  | Xia *et al.* 2013 |
| Atlantic salmon | *Salmo salar* | SC | U | M | M | C | N | 10.96 | 0.19 | Salmon feed | F | C | N | 5.95 | 0.32 | 0.08 | 5.01 |  | Dempson & Power 2004 |
| Australian mado | *Atypichthys strigatus* | C | U | M | M | N | N | 12.1 | 0.40 | Commercial flake food | T | N | N | 9.80 | 0.50 | 0.20 | 2.3 |  | Gaston *et al.* 2004 |
| Japanese bass | *Lateolabrax japonicas* | C | E | M | M | N | N | 10.53 | 0.25 | Control diet | F | N | N | 7.41 | 0.55 | 0.16 | 3.12 |  | Suzuki *et al.* 2005 |
| Japanese bass | *Lateolabrax japonicus* | C | E | M | M | N | N | 16.17 | 0.25 | Formulated | F | N | N | 13.76 | 0.46 | 0.16 | 2.41 |  | Suzuki *et al.* 2005 |
| Atlantic salmon | *Salmo salar* | C | E | M | M |  |  | 10.65 |  | Formulated | F |  |  | 8.15 |  | 0.30 | 2.50 |  | Trueman *et al.* 2005 |
| Salt marsh | *Fundulus heteroclitus* | C | E | M | M | N | N | 14.6 | 0.10 | Water soaked tuna | T |  |  | 15 |  | 0.10 | -0.40 |  | Logan *et al.* 2006 |
| Pacific herring | *Clupea pallasi* | C | E | M | M | N | N | 12.75 | 0.45 | Non enriched diet | F | N | N | 8 | 0.87 | 0.20 | 4.75 |  | Miller 2000 |
| Sea bass | *Dicentrarchus labrax* | C | E | M | M | N | N | 17.83 | 0.29 | Sandeel | T | N | N | 14 | 0.68 | 0.20 | 3.83 |  | Sweeting *et al.* 2007 |
| Sea bass | *Dicentrarchus labrax* | C | E | M | M | N | N | 17.48 | 0.37 | Dab muscle | T | N | N | 13.5 | 0.69 | 0.20 | 3.98 |  | Sweeting *et al.* 2007 |
| Bluefin tuna | *Thunnus thynnus* | C | U | M | M | Y | N | 11.51 |  | Fish | T | Y | N | 9.77 |  |  | 1.81 |  | Varela *et al.* 2011 |
| Black bream | *Acanthopagrus butcheri* | C | E | M | M | N | N | 14.60 |  | Hatchery feed | F | N | N | 9.6 | 0.10 | 0.18 | 5.00 |  | Bloomfield *et al.* 2011 |
| Black bream | *Acanthopagrus butcheri* | C | E | M | M | N | N | 13.85 |  | Fishmeal | F | N | N | 8.80 | 0.2 | 0.18 | 5.05 |  | Bloomfield *et al.* 2011 |
| Black bream | *Acanthopagrus butcheri* | C | E | M | M | N | N | 11.8 |  |  | F | N | N | 1.40 | 0.30 | 0.18 | 10.40 |  | Bloomfield *et al.* 2011 |
| Sand tiger / lemon | *Carcharias taurus / Negaprion brevirostris* | SC | E | M | M |  |  | 15.13 |  | Fish | T |  |  | 12.77 |  | 0.14 | 2.37 |  | Hussey *et al.* 2010 |
| Leopard | *Triakis semifasciata* | C | E | M | M | Y | N | 17.00 | 0.40 | Cephalopod (squid) | T | Y | Y | 13.30 | 0.70 | 0.20 | 3.7 |  | Kim *et al.* 2012a |
| Atlantic cod | *Gadus morhua* | C | U | M | M | N | N | 15.98 |  |  | T | N | N | 10.88 | 1.10 | 0.20 | 5.1 | 0.8 | Ankjaero *et al.* 2012 |
| Atlantic cod | *Gadus morhua* | C | U | M | M | N | N | 15.09 |  |  | T | N | N | 12.49 | 0.75 | 0.20 | 2.6 | 0.6 | Ankjaero *et al.* 2012 |
| Atlantic cod | *Gadus morhua* | C | U | M | M | N | N | 16.33 |  |  | T | N | N | 15.57 | 0.20 | 0.20 | 0.76 | 0.5 | Ankjaero *et al.* 2012 |
| European seabass | *Dicentrarchus labrax* | C | E | M | M | C | N | 16.50 | 0.37 |  | T | C | N | 12.18 | 1.01 |  | 4.32 |  | Barnes *et al.* 2007 |
| Summer flounder | *Paralichthys dentatus* | C | U | M | M | N | N | 8.80 |  |  | T | N | N | 3.71 | 0.48 |  | 5.09 |  | Buchheister & Latour 2010 |
| Salt marsh killifish | *Fundulus heteroclitus* | C | U | M | M |  |  | 15.56 |  |  | T | N | N | 11.60 |  |  | 3.96 |  | Eldson *et al.* 2010 |
| Salt marsh killifish | *Fundulus heteroclitus* | C | U | M | M |  |  | 9.36 |  |  | F | N | N | 1.00 |  |  | 8.36 |  | Eldson *et al.* 2010 |
| Salt marsh killifish | *Fundulus heteroclitus* | C | U | M | M |  |  | 15.00 |  |  | F | N | N | 11.80 |  |  | 3.20 |  | Eldson *et al.* 2010 |
| Salt marsh killifish | *Fundulus heteroclitus* | C | U | M | M |  |  | 16.93 |  |  | T | N | N | 13.70 |  |  | 3.23 |  | Eldson *et al.* 2010 |
| Salt marsh killifish | *Fundulus heteroclitus* | C | U | M | M |  |  | 15.43 |  |  | T | N | N | 11.20 |  |  | 4.23 |  | Eldson *et al.* 2010 |
| Bluefin tuna | *Thunnus orientalis* | C | E | M | M | C | N | 15.80 |  |  | U | C | N | 13.90 | 0.70 |  | 1.90 | 0.40 | Madigan *et al.* 2012 |
| Large spotted dogfish | *Scyliorhinus stellaris* | C | E | M | M | Y | N | 15.64 |  |  | T | N | N | 17.45 | 0.27 | 0.15 | -1.81 |  | Caut *et al.* 2013 |
| Large spotted dogfish | *Scyliorhinus stellaris* | C | E | M | M | Y | N | 13.21 |  |  | T | N | N | 9.72 | 0.24 | 0.15 | 3.49 |  | Caut *et al.* 2013 |
| Atlantic Bluefin tuna | *Thunnus thynnus* | C | U | M | M |  |  | 10.15 |  |  | T |  |  | 8.78 |  | 0.03 | 1.37 |  | Varela *et al.* 2012 |
| Sand goby | *Pomatoschistus minutus* | C | U | M | M | N | N | 13.20 | 0.20 |  | F | N | N | 9.14 | 0.22 | 0.2 | 4.06 |  | Guelinckx *et al.* 2007 |
| Leopard | *Triakis semifasciata* | C | E | M | M | Y | N | 13.40 |  |  | T | Y | Y | 7.90 | 0.40 | 0.20 | 5.50 | 0.40 | Kim *et al.* 2012 |
| Leopard | *Triakis semifasciata* | C | E | M | M | N | N | 8.90 |  |  | F | N | N | 6.60 | 0.20 | 0.16 | 2.30 |  | Malpica-Cruz *et al.* 2011 |

**REFERENCES:**

Ankjaero, T., Christensen, J.T. & Gronkjaer, P. (2012). Tissue-specific turnover rates and trophic enrichment of stable N and C isotopes in juvenile Atlantic cod *Gadus morhua* fed three different diets. *Mar. Ecol. Prog. Ser.*, 461, 197-209.

Barnes, C., Sweeting, C.J., Jennings, S., Barry, J.T. & Polunin, N.V.C. (2007). Effect of temperature and ration size on carbon and nitrogen stable isotope fractionation. *Funct. Ecol*., 21, 356-362.

Buchheister, A. & Latour, R.J. (2010). Turnover and fractionation of carbon and nitrogen stable isotopes in tissues of a migratory coastal predator, summer flounder (*Paralichthys dentatus*). *Can. J., Fish. Aquat. Sci.*, 67, 445-461.

Bloomfield, A.L., Elsdon, T.S., Walther, B.D., Gier, E.J. & Gillanders, B.M. (2011). Temperature and diet affect carbon and nitrogen isotopes of fish muscle: can amino acid nitrogen isotopes explain effects? *J. Exp. Mar. Biol. Ecol.*, 399, 48-59.

Caut, S., Jowers, M.J., Michel, L., LePoint, G. & Fisk, A.T. (2013). Diet- and tissue-specific isotopic incorporation in sharks: applications in a North Sea mesopredator. *Mar. Ecol. Prog. Ser.*, 492, 185-198.

Colborne, S.F. & Robinson, B.W. (2013). Effect of nutritional condition on variation in δ^13^C and δ^15^N stable isotope values in Pumpkinseed sunfish (*Lepomis gibbosus*) fed different diets. *Environ. Biol. Fish*, 96, 543-554.

Dempson, J. & Power, M. (2004). Use of stable isotopes to distinguish farmed from wild Atlantic salmon, *Salmo salar*. *Ecol. Freshw. Fish*, 13, 176-184.

Eldson, T.S., Ayvazian, S., McMahon, K.W. & Thorrold, S.R. (2010). Experimental evaluation of stable isotope fractionation in fish muscle and otoliths. *Mar. Ecol. Prog. Ser*., 408, 195-205.

Enyidi, U., Kiljunen, M., Jones, R.I. & Pirhonen, J. (2013). Nutrient assimilation by first-feeding African catfish, *Clarias gariepinus*, assessed using stable isotope analysis*. J. World Aquacult. Soc*., 44, 161-172.

Estep, M.L. & Vigg, S. (1985). Stable carbon and nitrogen isotope tracers of trophic dynamics in natural populations and fisheries of the Lahontan Lake system, Nevada. *Can. J. Fish. Aquat. Sci.*, 42, 1712-1719.

Gaston, T.F., Kostoglidis, A. & Suthers, I.M. (2004). The ^13^C, ^15^N and ^34^S signatures of a rocky reef planktivorous fish indicate different coastal discharges of sewage. *Mar. Freshw. Res*., 55, 689-699.

German, D.P. & Miles, R.D. (2010). Stable carbon and nitrogen incorporation in blood and fin tissue of the catfish *Pterygoplichthys disjunctivus* (Siluriformes, Loricariidae). *Environ. Biol. Fishes*, 89, 117-133.

Guelinckx, J., Maes, J., Van Den Driessche, P., Geysen, B., Dehairs, F. & Ollevier, F. (2007). Changes in δ^13^C and δ^15^N in different tissues of juvenile sand goby *Pomatoschistus minutus*: a laboratory diet-switch experiment. *Mar. Ecol. Prog. Ser*., 341, 205-215.

Harvey, C.J., Hanson, P.C., Essington, T.E., Brown, P.B. & Kitchell, J.F. (2002). Using bioenergetics models to predict stable isotope ratios in fishes. *Can. J. Fish. Aquat. Sci*., 59, 115-124.

Heady, W.N. & Moore, J.W. (2013). Tissue turnover and stable isotope clocks to quantify resource shifts in anadromous rainbow trout. *Oecologia*, 172, 21-34.

Hesslein, R.H., Hallard, K.A. & Ramlal, P. (1993). Replacement of sulfur, carbon, and nitrogen in tissue of growing broad whitefish (*Coregonus nasus*) in response to a change in diet traced by δ^34^S, δ^13^C, and δ^15^N. *Can. J. Fish. Aquat. Sci*., 50, 2071-2076.

Hussey, N.E., Brush, J., McCarthy, I.D. & Fisk, A.T. (2010). ^15^N and ^13^C diet–tissue discrimination factors for large sharks under semi-controlled conditions. *Comp. Biochem. Physiol. A*, 155, 445-453.

Kim, S.L., Casper, D.R., Galván-Magaña, F., Ochoa-Díaz, R., Hernández-Aguilar, S.B. & Koch, P.L. (2012a). Carbon and nitrogen discrimination factors for elasmobranch soft tissues based on a long-term controlled feeding study*. Environ. Biol. Fishes*, 95, 37-52.

Kim, S.L., Martinez del Rio, C., Casper, D. & Koch, P.L. (2012). Isotopic incorporation rates for shark tissues from a long-term captive feeding study. *J. Exp. Biol.*, 215, 2495-2500.

Logan, J., Haas, H., Deegan, L. & Gaines, E. (2006). Turnover rates of nitrogen stable isotopes in the salt marsh mummichog, *Fundulus heteroclitus*, following a laboratory diet switch. *Oecologia*, 147, 391-395.

Madigan, D.J., Litvin, S.Y., Popp, B.N., Carlisle, A.B., Farwell, C.J. & Block, B.A. (2012). Tissue turnover rates and isotopic trophic discrimination factors in the endothermic teleost, Pacific Bluefin tuna (*Thunnus orientalis*). *PLoS ONE* 7(11): e49220. doi:10.1371/journal.pone.0049220

Malpica-Cruz, L., Herzka, S.Z., Sosa-Nishizaki, O. & Pablo Lazo, J. (2012). Tissue-specific isotope trophic discrimination factors and turnover rates in a marine elasmobranch: empirical and modeling results. *Can. J. Fish. Aquat. Sci.*, 69, 551-564.

Miller, T.W. (2000). Tissue-specific response of δ15 N in adult Pacific herring (*Clupea pallasi*) following an isotopic shift in diet. *Environ. Biol. Fish,* 76, 177-189.

Peterson, B.J. & Howarth, R.W. (1987). Sulfur, carbon and nitrogen isotopes used to trace organic matter flow in the salt-marsh estuaries of Sapelo Island, Georgia. *Limnol. Oceanogr*., 32, 1195-1213.

Pinnegar, J.K. & Polunin, N.V.C. (1999). Differential fractionation of δ^13^C and δ^15^N among fish tissues: implications for the study of trophic interactions. *Funct. Ecol.*, 13, 225-231.

Suzuki, K.W., Kasai, A., Nakayama, K. & Tanaka, M. (2005). Differential isotopic enrichment and half-life among tissues in Japanese temperate bass (*Lateolabrax japonicus*) juveniles: implications for analyzing migration*. Can. J. Fish. Aquat. Sci*., 62, 671-678.

Sweeting, C., Barry, J., Barnes, C., Polunin, N. & Jennings, S. (2007). Effects of body size and environment on diet-tissue δ^15^N fractionation in fishes. *J. Exp. Mar. Biol. Ecol*., 340, 1-10.

Trueman, C.N., McGill, R.A. & Guyard, P.H. (2005). The effect of growth rate on tissue-diet isotopic spacing in rapidly growing animals. An experimental study with Atlantic salmon (*Salmo salar*). *Rapid Commun. Mass Spectrom.*, 19, 3239-3247.

Varela, J.L., Larrañaga, A. & Medina, A. (2011). Prey-muscle carbon and nitrogen stable-isotope discrimination factors in Atlantic bluefin tuna (*Thunnus thynnus*). *J. Exp. Mar. Biol. Ecol*., 406, 21-28.

Varela, J.L., de la Gandara, F., Ortega, A. & Medina, A. (2012). ^13^C and ^15^N analysis in muscle and liver of wild and reared young-of-the-year (YOY) Atlantic Bluefin tuna. *Aquaculture*, 354-355, 17-21.

Vollaire, Y., Banas, D., Thomas, M. & Roche, H. (2007). Stable isotope variability in tissues of the Eurasian perch *Perca fluviatilis*. *Comp. Biochem. Physiol. A*, 148, 504-509.

Xia, B., Gao, Q.F., Li, H., Dong, S.L. & Wang, F. (2013). Turnover and fractionation of nitrogen stable isotope in tissues of grass carp *Ctenopharyngodon idellus.* *Aquaculture Environ. Inter.*, 177, 177-186.
