# Supplementary material for: Rescaling the trophic structure of marine food webs
Source: Ecol Lett. 2013 Dec 6;17(2):239–50. doi: 10.1111/ele.12226 (PMC3912912; doi:10.1111/ele.12226)
Supplement: Supplementary file 2 [file ele0017-0239-sd2.docx]

**Supplementary Material Table S2:** Details of species sampled from KwaZulu-Natal, South Africa and Cumberland Sound, Canadian Arctic included in the trophic framework analyses.

| **SPECIES** |  |  |  |  |  |  | |  | |  |
| --- | --- | --- | --- | --- | --- | --- | --- | --- | --- | --- |
| **Common name** | **Scientific**  **name** | **n** | **Length (mm)^A^** | **Sample location^B^** | **Sampling date** | **Sampling method^C^** | | **Habitat^D^** | | **Diet TP^E^** |
| **TELEOST** | | | | | | | | | | |
| **KwaZulu-Natal, South Africa** | | | | | | | | | | |
| Salema | *Sarpa salpa* | 3 | 226.7 ± 23.6  (200-245) | Durban – ORI beach, SA | 01/08/2008 | RF/S-C | Benthopelagic | | 2.14 ± 0.14 | |
| White seabream | *Diplodus sargus* | 6 | 194 ± 81.8  (115-300) | Durban – ORI, SA | 28/07/2008  01/08/2008 | RF/S-C | Demersal | | 3.24 ± 0.46 | |
| Natal pandora | *Pagellus natalensis* | 4 | 154.3 ± 5.7  (148-160) | Durban – ORI beach, SA | 28/07/2008 | RF/S-C | Demersal | | 3.34 ± 0.39 | |
| Flathead grey mullet | *Mugil cephalus* | 1 | 400 | Durban – ORI beach, SA | 01/08/2008 | RF/S-C | Benthopelagic | | 2.13 ± 0.18 | |
| Garrick | *Lichia amia* | 1 | 1040 | Durban – ORI shelf, SA | 13//07/2008 | OSP-C | Pelagic/neritic | | 4.5 ± 0.8 | |
| African scad | *Trachurus delagoa* | 2 | 191, 235 | Durban – ORI beach/ Park Rynie, SA | 05/07/2008  28/07/2008 | RF/S-C | Benthopelagic | | 3.73 ± 0.57 | |
| Bluefish | *Pomatomus saltatrix* | 5 | 270 ± 64.4  (190-370) | Thukela Banks, SA | 27/05/2006  30/05/2006 | TR | Pelagic | | 4.5 ± 0.55 | |
| Common ponyfish | *Leiognatus equula* | 5 | 144 ± 45.1  (110-220) | Thukela Banks, SA | 29/05/2006  01/06/2006 | TR | Demersal | | 2.45 ± 0.10 | |
| Small spotted grunter | *Pomadasys commersonni* | 5 | 416 ±  166.8  (300-710 | Thukela Banks, SA | 31/05/2006 | TR | Reef associated | | 3.48 ± 0.59 | |
| Slinger seabream | *Chrysoblephus puniceus* | 5 | 333 ± 16.0  (310-350) | Mttentu River, SA | 05/07/2008 | RF/S-C | Demersal | | 3.5 ± 0.46 | |
| Yellowfin tuna | *Thunnus albacares* | 1 | 1500 | Durban, SA | 15/07/2008 | RF/S-C | Pelagic | | 4.34 ± 0.71 | |
| Tigertooth croaker | *Otolithes ruber* | 5 | 294 ± 62.3  (230-390) | Thukela Banks, SA | 27/05/2006 | TR | Benthopelagic | | 3.6 ± 0.59 | |
| Natal snoek | *Scomberomoros plurilineatus* | 1 | 680 | Durban – ORI beach, SA | 15/07/2008 | OSP-C | Pelagic/Neritic | | 4.24 ± 0.77 | |
| Largehead hairtail | *Trichiurus lepturus* | 5 | 618 ± 44.4  (570-670) | Thukela Banks, SA | 27/05/2006 | TR | Benthopelagic | | 4.45 ± 0.77 | |
| Chub mackerel | *Scomber japonicus* | 5 | 229 ± 6.5  (220-235) | South Durban, SA | 26/06/2008 | RF/S-C | Pelagic | | 3.09 ± 0.43 | |
| Fourline tonguesole | *Cynoglossus attenuatus* | 5 | 242 ± 23.9  (220-280) | Thukela Banks, SA | 29/05/2006 | TR | Demersal | | 3.5 ± 0.37 | |
| Cape knifejaw | *Oplegnathus conwayi* | 1 | 435 | Durban – ORI beach, SA | 13/07/2008 | OSP-C | Reef associated | | 2.7 ± 0.24 | |
| Indian scad | *Decapterus russelli* | 1 | 187 | Durban – ORI beach, SA | 28/07/2008 | RF/S-C | Benthopelagic | | 3.69 ± 0.58 | |
| Grooved mullet | *Liza dumerilii* | 5 | 166.6 ± 73.5  (78-240) | Tinley Manor / Durban – ORI beach, SA | 15/07/2008  01/08/2008 | RF/S-C | Demersal | | 2.68 ± 0.32 | |
| Olive grunt | *Pomadasys olivaceus* | 5 | 204 ± 20.7  (170-220) | Thukela Banks, SA | 27/05/2006 | TR | Reef associated | | 2.64 ± 0.14 | |
| Slender baardman | *Umbrina rhonchus* | 2 | 775 | Durban – ORI beach / Richards Bay, SA | 05/07/2008  13/07/2008 | OSP-C | Demersal | | 3.38 ± 0.42 | |
| South African sardine | *Sardinops sagax* | 5 | 180 ± 12.2  (160-193) | Warner Beach, SA | 11/07/2008 | TR | Pelagic/Neritic | | 2.43 ± 0.12 | |
| Pugnose ponyfish | *Secutor insidiator* | 5 | 98 ± 4.5  (90-100) | Thukela Banks, SA | 29/05/2006 | TR | Demersal | | 2.84 ± 0.27 | |
| Squaretail kob | *Argyrosomus thorpei* | 4 | 357.5 ± 37.7  (240-260) | Thukela Banks, SA | 27/05/2006  30/05/2006 | TR | Demersal | | 3.97 ± 0.67 | |
| Sharpfin barracuda | *Sphyraena acutipinnis* | 4 | 252.5 ± 9.6  (240-260) | Thukela Banks, SA | 01/06/2006 | TR | Reef associated | | 4.5 ± 0.8 | |
| Blackmouth croaker | *Atrobucca nibe* | 5 | 140 ±18.7  (120-160) | Thukela Banks, SA | 27/05/2006 | TR | Demersal | | 3.64 ± 0.53 | |
| Striped grunt | *Pomadasys striatus* | 5 | 156 ± 11.4  (140-170) | Thukela Banks, SA | 27/05/2006 | TR | Benthopelagic | | 3.39 ± 0.47 | |
| Barbel | *Galeichthys sp.* | 4 | 342.5 ± 125.5  (190-480) | Thukela Banks, SA | 27/05/2006  29/05/2006  30/05/2006  31/05/2006 | TR | Demersal | | 3.5 ± 0.5 | |
| Orangemouth anchovy | *Thryssa vitrirostris* | 5 | 184 ± 16.7  (170-210) | Thukela Banks, SA | 27/05/2006  29/05/2006 | TR | Pelagic/Neritic | | 3.31± 0.46 | |
| Small kob | *Johnius dorsalis* | 5 | 180 ± 15.8  (160-200) | Thukela Banks, SA | 27/05/2006 | TR | Demersal | | 3.33± 0.40 | |
| Bellfish | *Johnius fuscolineatus* | 5 | 228 ± 53.6  (170-300) | Thukela Banks, SA | 27/05/2006 | TR | Benthopelagic | | n/a | |
| Shad | *Pellona ditchella* | 1 | 150 | Thukela Banks, SA | 31/05/2006 | TR | Pelagic/Neritic | | n/a | |
| Barracuda | *Sphyraena putnamiae* | 1 | 245 | Park Rynie, SA | 05/07/2008 | RF/S-C | Reef associated | | 4.47 ± 0.79 | |
| **Cumberland Sound, The Canadian Arctic** | | | | | | | | | | |
| Capelin | *Mallotus villosus* | 7 | 135 ± 12  (120-150) | Cumberland Sound, CA | 31/07/2008 | DN | Pelagic | | 3.2 ± 0.1 | |
| Herring | *Clupea harengus* | 1 | 278 | Cumberland Sound, CA | 09/08/2007 | GN | Pelagic | | 3.6 ± 0.5 | |
| Sculpin | *Myoxocephalus scorpius* | 18 | 262 ± 50  (180-335) | Cumberland Sound, CA | 10-17/08/2009 | GN | Benthic | | 3.9 | |
| Arctic char | *Salvelinus alpinus* | 10 | 567 ± 102 (340-680) | Cumberland Sound, CA | 30/07/2008 | GN | Pelagic | | 4.4 ± 0.1 | |
| Greenland halibut | *Reinhardtius hippoglossoides* | 76 | 629 ± 95  (480-885) | Cumberland Sound, CA | 2008/2009 | LL | Benthic | | 4.4 ± 0.2 | |
| **ELASMOBRANCH** | | | | | | | | | | |
| **KwaZulu-Natal, South Africa** | | | | | | | | | | |
| Milk | *Rhizoprionodon acutus* | 3 | 740 ± 26.5  (710-760) | KwaZulu-Natal, SA | 2005/2006 | NET | Marine/ Brackish/ Benthopelagic / Amphidromous | | 4.3 ± 0.1  (4.1) | |
| Copper | *Carcharhinus brachyurus* | 5 | 1972 219.9  (1700-2160) | KwaZulu-Natal, SA | 2006/2010 | NET | Marine/ Brackish/ Reef associated/ Oceanodromous | | 4.3/ 4.5  (4.2) | |
| Spinner | *Carcharhinus brevipinna* | 24 | 1293.5 ± 478.7  (570-1932) | KwaZulu-Natal, SA | 2006/2007 | NET | Reef associated/ Oceanodromous | | (4.2) | |
| Bull | *Carcharhinus leucas* | 12 | 1579 ± 310.8  (790-2080) | KwaZulu-Natal, SA | 2005/2006  2007/2009 | NET | Marine/ freshwater/ Brackish/ Oceanodromous | | (4.3) | |
| Tiger | *Galeocerdo cuvier* | 18 | 1952.9 ± 356.2  (1600-2390) | KwaZulu-Natal, SA | 2006/2007  2008/2009 2010 | NET | Marine/ Brackish/ Benthopelagic / Oceanodromous | | 3.8/ 4.5  (4.1) | |
| Smooth | *Sphyrna zygaena* | 12 | 1002 ± 121.6  (698-1158) | KwaZulu-Natal, SA | 2005/2006 2007 | NET | Marine/ Brackish/ Pelagic-oceanic/ Oceanodromous | | 4.5/ 5.4  (4.2) | |
| Scalloped | *Sphyrna lewini* | 39 | 1258.8 ± 598.2  (380-2234) | KwaZulu-Natal, SA | 2005/2006 2007/2010 | NET | Marine/ Brackish/ Pelagic-oceanic/ Oceanodromous | | 4.0/ 4.5  (4.1) | |
| Blacktip | *Carcharhinus limbatus* | 32 | 1531.9 ± 202.2  (1126-1858) | KwaZulu-Natal, SA | 2005/2006 2007 | NET | n/a | | 4.2  (4.5) | |
| Dusky | *Carcharhinus obscurus* | 64 | 1643.3 ± 729.3  (700-2800) | KwaZulu-Natal, SA | 2005/2006 2007/2008 | NET | n/a | | 4.2 ± 0.4  (4.2) | |
| Sandbar | *Carcharhinus plumbeus* | 6 | 1243 ± 165.5  (930-1390) | KwaZulu-Natal, SA | 2005/2006 2007 | NET | n/a | | 4.5  (4.1) | |
| Sandtiger | *Carcharias taurus* | 30 | 1910.8 ± 145.5  (1490-2092) | KwaZulu-Natal, SA | 2005/2006  2007/2008 | NET | n/a | | 4.4  (4.5) | |
| Java | *Carcharhinus amboinensis* | 9 | 1310.4 ± 141.4  (1062-1520) | KwaZulu-Natal, SA | 2005/2006 2007 | NET | Marine/ Brackish/ Reef associated | | (4.3) | |
| Thintail thresher | *Alopias vulpinus* | 1 | 2000 | KwaZulu-Natal, SA | 2006 | NET | n/a | | 4.5  (4.2) | |
| Shortfin Mako | *Isurus oxyrinchus* | 4 | 2032 ± 282.1  (1610-2200) | KwaZulu-Natal, SA | 2005/2006 2007 | NET | n/a | | 4.5/ 5.4  (4.3) | |
| Whale | *Rhincodon typus* | 3 | 68400 ± 2506  (66000-71000) | KwaZulu-Natal, SA | 2008/2009 | STRAND | n/a | | (3.5) | |
| White | *Carcharodon carcharias* | 37 | 2176.5 ± 533.6  (1600-3630) | KwaZulu-Natal, SA | 2005/2006 2007/2008 2009 | NET | n/a | | 4.9  (4.5) | |
| Devil ray | *Mobula* sp*.* | 9 | 789.1 ± 209.5  (580-1140) | KwaZulu-Natal, SA | 2008/2009 2010 | NET | n/a | | n/a | |
| Giant guitarfish | *Rhynchobatus djiddensis* | 3 | 1543.3 ± 47.3  (1490-1580) | KwaZulu-Natal, SA | 2009 | NET | Marine/Brackish/Reef associated | | 3.6 | |
| Bull ray | *Pteromylaeus bovinus* | 8 | 1022 ± 245.7  (750-1530) | KwaZulu-Natal, SA | 2008/2010 | NET | Marine/Brackish/Benthopelagic | | n/a | |
| **Cumberland Sound, The Canadian Arctic** | | | | | | | | | | |
| Arctic skate | *Amblyraja hyperborea* | 18 | 597 ± 67  (480-700) | Cumberland Sound, CA | 2008/2009 | LL | Benthic | | 4.3 | |
| Greenland shark | *Somniosus microcephalus* | 63 | 2962 ± 342  (2220-3780) | Cumberland Sound, CA | 2008/2009 | LL | Benthopelagic | | 4.2  (4.2) | |

^A^ Length measurements for all teleost fish are total length, for elasmobranchs are precaudal length (PCL) with the exception of the devil ray and bull ray (disc width - DW) and the Greenland shark and Arctic skate (total length).

^B^ Sampling locations equate to two study sites, KwaZulu-Natal, South Africa (SA) and Cumberland Sound, Canadian Arctic (CA).

^C^ Sampling methods were as follows: TR – shallow water prawn trawl; RF-S-C – organized recreational fishermen and scientific catches; OSP-C – organized spearfishermen catches; NET – beach protection gill net (KwaZulu-Natal Sharks Board); STRAND – natural animal stranding, DN – scientific dip net; GN – scientific gill net; LL – scientific long-line.

^D^ Habitat information were derived from Fishbase.org

^E^ For teleost fish trophic position (TP) is the mean (± SD) of TP estimates calculated by Fishbase.org. For elasmobranchs, TP is the mean (± SD) of TP estimates calculated by Fishbase.org plus in parentheses that calculated from stomach contents by Cortes (1999)
