# Supplementary material for: Rescaling the trophic structure of marine food webs
Source: Ecol Lett. 2013 Dec 6;17(2):239–50. doi: 10.1111/ele.12226 (PMC3912912; doi:10.1111/ele.12226)
Supplement: Supplementary file 3 [file ele0017-0239-sd3.docx]

**Supplementary Material S3**

*Stable isotope analysis and precision*

Stable isotope concentrations are expressed in delta notation (δ^15^N in parts per thousand) as:

$\delta X= \left[ \left( \frac{R_{Sample}}{R_{Standard}} \right)-1 \right]*1000$ [1]

where X is ^15^N or ^13^C and R is the ratio ^15^N/^14^N or ^13^C/^12^C. The standard reference material was Pee Dee Belemnite for CO_2_ and atmospheric nitrogen for N_2_. The analytical precision based on NIST 8414 and internal laboratory fish muscle standard (n=111) for δ^15^N were 0.16‰ and 0.23‰, respectively, and for δ^13^C were both 0.07‰. Additional certified standards (NIST 8542 and NIST 8542; n=3) generated values that were within 0.01‰ and 0.07‰ for δ^15^N and δ^13^C.
