# Supplementary material for: Rescaling the trophic structure of marine food webs
Source: Ecol Lett. 2013 Dec 6;17(2):239–50. doi: 10.1111/ele.12226 (PMC3912912; doi:10.1111/ele.12226)
Supplement: Supplementary file 4 [file ele0017-0239-sd4.docx]

**Supplementary Material S4**

*Dietary δ^15^N value-dependent Δ^15^N model*

The negative relationship between dietary δ^15^N and consumer δ^15^N found in the meta-analysis implies: (a) that the diet tissue fractionation factor (Δ^15^N) decreases with increasing dietary δ^15^N; (b) that there is a dietary δ^15^N value at which Δ ^15^N is zero; and (c) that there is a point of attraction in the relationship between dietary δ^15^N and consumer δ^15^N toward which consumer δ^15^N will equilibrate even if trophic level (TL) increases.

If true, these three results suggest a point at which the amount of ^15^N, and ^14^N, taken up by a consumer equals the amount at which ^15^N, and ^14^N, is eliminated from their tissues, and thus no net change in δ^15^N between diet and consumer. Faster breakdown of ^14^N- compared to ^15^N-amino acids has been the proposed mechanism for enrichment of ^15^N in consumers (Macko *et al*. 1996, 1997), but this mechanism has ignored the source pool (i.e., in the food and consumer) of nitrogen isotopes. The rate at which nitrogen isotopes are taken-up and eliminated is likely constant under normal physiological states, since it is believed that the difference in ^14^N and ^15^N is related to chemical characteristics of the isotopes and not enzymatic characteristics (Macko *et al*. 1996, 1997). Thus, at lower δ^15^N values the relative source pools of ^14^N and ^15^N favor greater ^15^N incorporation and an increase in δ^15^N value in the consumer versus food.

A model describing such a relationship requires knowing the δ^15^N value at the base of the food web (δ^15^N_base_), the value of dietary δ^15^N at which ^15^N incorporation and ^15^N elimination are equal (δ^15^N_lim_), as well as the value at which the ratio between ^15^N incorporation and ^15^N elimination changes relative to dietary δ^15^N averaged across the food-web (*k*).

$\delta^{15}N_{TP}=\delta^{15}N_{\lim}-(\delta^{15}N_{\lim}-\delta^{15}N_{base})e^{-k*TP}$. (S1)

As for conventional additive food webs, δ^15^N_base_ is estimable from appropriate primary or secondary consumers that characterize δ^15^N at the base of the food web, typically zooplanktivores. Both δ^15^N_lim_ and *k* on the other hand are estimable from the linear relationship between dietary δ^15^N and the D^15^N of their consumers:

$\Delta^{15}N=\beta_{0}+\beta_{1}\delta^{15}N_{diet}$ (S2)

where

 (S3)

and

$\delta^{15}N_{\lim}=\frac{-\beta_{0}}{\beta_{1}}$. (S4)

With these estimates in hand from the meta-analysis, and rearranging S1, we can estimate TP for a given δ^15^N_TP_ signature as:

$TP=\frac{\log(\delta^{15}N_{\lim}-\delta^{15}N_{base})-\log(\delta^{15}N_{\lim}-\delta^{15}N_{TP})}{k}$. (S5)

**REFERENCES:**

Macko, S. A., Fogel-Estep, M.L., Engel, M.H. & Hare, P.E. (1986). Kinetic fractionation of nitrogen isotopes during amino acid transamination. *Geochem. Cosmochim. Ac.*, 50, 2143–2146.

Macko, S. A., Fogel-Estep, M.L., Engel, M.H., & Hare, P.E. (1987). Isotopic fractionation of nitrogen and carbon in the synthesis of amino acids by microorganisms. *Chem. Geol.*, 65, 79–92
