# Supplementary material for: Rescaling the trophic structure of marine food webs
Source: Ecol Lett. 2013 Dec 6;17(2):239–50. doi: 10.1111/ele.12226 (PMC3912912; doi:10.1111/ele.12226)
Supplement: Supplementary file 5 [file ele0017-0239-sd5.docx]

**Supplementary Material S5**

*Code for Meta-analytical Model and Scaled Δ^15^N framework based on a dietary δ^15^N value-dependent Δ^15^N model*

Model is in Python 2.7.2 and PyMC 2.2

import pdb

import numpy as np

from pymc import *

from data import *

# Meta-analysis of experimental diet-tissue discrimination factor relationship between consumers and diets

## Variables

# 'diet' is the reported mean d15N value of the diet

# 'diet_sd' is the reported d15N standard deviation of the diet, which is assumed known

# 'consumer' is the reported mean d15N for the consumer

# 'consumer_sd' is the reported d15N standard deviation of the consumer, which is assumed known

# Meta-analysis slope

beta0 = Normal('beta0', mu=0.0, tau=0.001, value=10.0)

# Meta-analysis intercept

beta1 = Normal('beta1', mu=0.0, tau=0.001, value=-0.2)

# Latent diet values

theta_d = Normal('theta_d', mu=0.0, tau=0.001, value=np.ones(nobs)*mean(diet))

Zi = Normal('Zi', mu=theta_d, tau=diet_sd**-2, value=diet, observed=T)

# Linear model for relationship to consumer value

theta_mu = Lambda('theta_mu', lambda b0=beta0, b1=beta1, td=theta_d: b0+b1*td+td)

# Process error model

pro_sd = Uniform('pro_sd', lower=0, upper=100)

pro_tau = Lambda('pro_tau', lambda sd=pro_sd: sd**-2)

theta_c = Normal('theta_c', mu=theta_mu, tau=pro_tau)

# Consumer error model

Yi = Normal('Yi', mu=theta_c, tau=consumer_sd**-2, value=consumer, observed=T)

# Linear estimates for dtdf

d15N_lim = Lambda('d15N_lim', lambda b0=beta0, b1=beta1: -(b0/b1))

k = Lambda('k', lambda b0=beta0, dlim=d15N_lim: -np.log((b0-dlim)/-dlim))

# South Africa Baseline

SA_B2 = Uniform('SA_B2', lower=0.1, upper=100, value=np.mean(sa_d15N[sa_base=='2']))

SA_B3 = Uniform('SA_B3', lower=0.1, upper=100, value=np.mean(sa_d15N[sa_base=='3']))

SA_sd = Uniform('SA_sd', lower=0, upper=100, value=(2,2))

SA_tau = Lambda('SA_tau', lambda sd=SA_sd: sd**-2)

SA_d15N_base2 = Normal('SA_d15N_base2', mu=SA_B2, tau=SA_tau[0], value=sa_d15N[sa_base=='2'], observed=T)

SA_d15N_base3 = Normal('SA_d15N_base3', mu=SA_B3, tau=SA_tau[1], value=sa_d15N[sa_base=='3'], observed=T)

# South Africa species TP estimates

mu_sa_C_d15N = Normal('mu_sa_C_d15N', mu=0.0, tau=0.1, value=np.ones(ncon_sa)*15)

SA_C_sd = Uniform('SA_C_sd', lower=0, upper=100, value=2)

SA_C_tau = Lambda('SA_C_tau', lambda sd=SA_C_sd: sd**-2)

zi_sa_C_d15N = Normal('zi_sa_C_d15N', mu=mu_sa_C_d15N[saIc], tau=SA_C_tau, value=sa_C_d15N, observed=T)

SA_TP_base2 = Lambda('SA_TP_base2', lambda dlim=d15N_lim, k=k, dbase=SA_B2, mud15N=mu_sa_C_d15N: (np.log(dlim-dbase)-np.log(dlim-mud15N))/k+2)

SA_TP2_spp = [Lambda('SA_TP2_%s'%sa_consumer[i], lambda tp=SA_TP_base2[i]: tp) for i in xrange(ncon_sa)]

SA_TP_base3 = Lambda('SA_TP_base3', lambda dlim=d15N_lim, k=k, dbase=SA_B3, mud15N=mu_sa_C_d15N: (np.log(dlim-dbase)-np.log(dlim-mud15N))/k+3)

SA_TP3_spp = [Lambda('SA_TP3_%s'%sa_consumer[i], lambda tp=SA_TP_base3[i]: tp) for i in xrange(ncon_sa)]

# South Africa individual TP estimates

SA_TP_ibase2 = Lambda('SA_TP_ibase2', lambda dlim=d15N_lim, k=k, dbase=SA_B2: (np.log(dlim-dbase)-np.log(dlim-sa_C_d15N))/k+2)

SA_TP_ibase3 = Lambda('SA_TP_ibase3', lambda dlim=d15N_lim, k=k, dbase=SA_B3: (np.log(dlim-dbase)-np.log(dlim-sa_C_d15N))/k+3)

# Cumberland Sound Baseline

CS_B2 = Uniform('CS_B2', lower=0.1, upper=100, value=np.mean(cs_d15N[cs_base=='2']))

CS_B3 = Uniform('CS_B3', lower=0.1, upper=100, value=np.mean(cs_d15N[cs_base=='3']))

CS_sd = Uniform('CS_sd', lower=0, upper=100, value=(2,2))

CS_tau = Lambda('CS_tau', lambda sd=CS_sd: sd**-2)

CS_d15N_base2 = Normal('CS_d15N_base2', mu=CS_B2, tau=CS_tau[0], value=cs_d15N[cs_base=='2'], observed=T)

CS_d15N_base3 = Normal('CS_d15N_base3', mu=CS_B3, tau=CS_tau[1], value=cs_d15N[cs_base=='3'], observed=T)

# Cumberland Sound TP estimates

CS_sd_TL = Uniform('CS_sd_TL', lower=0, upper=100, value=2)

CS_tau_TL = Lambda('CS_tau_TL', lambda sd=CS_sd_TL: sd**-2)

mu_cs_C_d15N = Normal('mu_cs_C_d15N', mu=0.0, tau=CS_tau_TL, value=np.ones(ncon_cs)*15)

CS_C_sd = Uniform('CS_C_sd', lower=0, upper=100, value=2)

CS_C_tau = Lambda('CS_C_tau', lambda sd=CS_C_sd: sd**-2)

zi_cs_C_d15N = Normal('zi_cs_C_d15N', mu=mu_cs_C_d15N[csIc], tau=CS_C_tau, value=cs_C_d15N, observed=T)

CS_TP_base2 = Lambda('CS_TP_base2', lambda dlim=d15N_lim, k=k, dbase=CS_B2, mud15N=mu_cs_C_d15N: (np.log(dlim-dbase)-np.log(dlim-mud15N))/k+2)

CS_TP2_spp = [Lambda('CS_TP2_%s'%cs_consumer[i], lambda tp=CS_TP_base2[i]: tp) for i in xrange(ncon_cs)]

CS_TP_base3 = Lambda('CS_TP_base3', lambda dlim=d15N_lim, k=k, dbase=CS_B3, mud15N=mu_cs_C_d15N: (np.log(dlim-dbase)-np.log(dlim-mud15N))/k+3)

CS_TP3_spp = [Lambda('CS_TP3_%s'%cs_consumer[i], lambda tp=CS_TP_base3[i]: tp) for i in xrange(ncon_cs)]

# Cumberland Sound individual TP estimates

CS_TP_ibase2 = Lambda('CS_TP_ibase2', lambda dlim=d15N_lim, k=k, dbase=CS_B2: (np.log(dlim-dbase)-np.log(dlim-cs_C_d15N))/k+2)

CS_TP_ibase3 = Lambda('CS_TP_ibase3', lambda dlim=d15N_lim, k=k, dbase=CS_B3: (np.log(dlim-dbase)-np.log(dlim-cs_C_d15N))/k+3)

# Calculate d15N for given discrete TLs

d15N_TL2_CS = Lambda('d15N_TL2_CS', lambda dlim=d15N_lim, dbase=CS_B2, k=k: dlim-(dlim-dbase)*np.exp(-k*TPx))

d15N_TL2_SA = Lambda('d15N_TL2_SA', lambda dlim=d15N_lim, dbase=SA_B2, k=k: dlim-(dlim-dbase)*np.exp(-k*TPx))

# Calculate d15N for given discrete TLs

d15N_TL3_CS = Lambda('d15N_TL3_CS', lambda dlim=d15N_lim, dbase=CS_B3, k=k: dlim-(dlim-dbase)*np.exp(-k*TPx))

d15N_TL3_SA = Lambda('d15N_TL3_SA', lambda dlim=d15N_lim, dbase=SA_B3, k=k: dlim-(dlim-dbase)*np.exp(-k*TPx))
