# Supplementary material for: Rescaling the trophic structure of marine food webs
Source: Ecol Lett. 2013 Dec 6;17(2):239–50. doi: 10.1111/ele.12226 (PMC3912912; doi:10.1111/ele.12226)
Supplement: Supplementary file 6 [file ele0017-0239-sd6.doc]

**Supplementary Material Table S6:** Summarized stomach contents of well-characterized lower trophic position (zooplanktivores) and higher trophic position (apex predators) species and primary/secondary piscivores in South Africa and the Canadian Arctic.

| **Species** | **Diet** | | | **Reference** | |
| --- | --- | --- | --- | --- | --- |
|  | **Trophic Functional group** | **Dominant prey items** |  | |  |
| **KWAZULU-NATAL, SOUTH AFRICA** | | | | | |
| **ZOOPLANKTIVORE** | | | | | |
| Longhorned /shorthfin devil ray  (*Mobula eregoodootenkee*, *Mobula* *kuhlii* [Msp]) | Zooplanktivore | Euphasiids, Nyctiphanes simplex, mysids | Brinton & Townsend (1980); Notarbartolo-di-Sciara (1988); De Silva-Dávila & Palmares-García, (2002) | |  |
| South African sardine  (*Sardinops sagax* [SO]) | Zooplanktivore | Calenoid, cyclopoid harpactacoid and eucalanoid copepods, phytoplankton (dinoflagellates) fish and crustacean eggs | Van der Lingen et al. (2010); Espinoza et al. (2009), Van der Lingen (200)2 | |  |
| Whale shark  (*Rhincodon typus* [RT]) | Zooplanktivore | Euphasiids, crab larvae, siphonophores, salps, sergestids, calenoid copepods, isopods, amphipods, stomatopods, coral spawn, chaetognaths, fish larvae, small squid and fish^A^ | Silas &Rajagopalan (1963), Taylor (1994); Taylor (1996,2007); Clark & Nelson (1997); Taylor & Pearce (1999); Heyman et al. (2001); Wilson & Newbound (2001); Duffy (2002); Jarman & Wilson (2004); Hacohen-Domene et al. (2006); Meekan et al. (2009); Motta et al. (2010) | |  |
| **PRIMARY PISCIVORE** | | | | | |
| Salema  (*Sarpa salpa* [SS]) | Herbivore | Plankton, algae and chironomid larvae | Bennett (1989); Havelange et al. (1997); Lechanteur & Griffiths (2003) | |  |
| Yellowfin tuna  (*Thunnus albacares* [TA]) | Primary Piscivore | Fishes, crustaceans, decapod and stomatopod larvae and adults, and squid | Smale (1986), Graham et al. (2006); Rohit et al. (2011); Rudershausen et al. (201); Kuhnert et al. (2012) | |  |
| Small spotted grunter (*Pomadasys commersonni* [PC]) | Primary Piscivore | Fishes, crustaceans (shrimps and prawns), mollusks | Van Der Westhuizen & Marais, (1977) | |  |
| Slinger seabream  (*Chrysoblephus puniceus* [CP]) | Primary Piscivore | Fishes, crustaceans, mollusks | Fischer at al. (1990) | |  |
| Small kob  (*Johnius dorsalis* [JD]) | Primary Piscivore |  |  | |  |
| Squaretail kob  (*Argyrosomus thorpei* [AT]) | Primary Piscivore | Fishes, crustaceans, cephalopods (squid) | Griffiths (1997) | |  |
| Olive grunt  (*Pomadasys olivaceus* [PO]) | Primary Piscivore | Fishes, molluscs, bivalves | Van der Elst & Adkin (1991) | |  |
| Striped grunt  (*Pomadasys striatum* [PS]) | Primary piscivore | Fishes, crustaceans (shrimps/crabs), mollusks, worms | Van der Elst (1986) | |  |
| Barbel  (*Galeichthys* sp. [Gsp]) | Primary Piscivore | Fishes, cephalopods, crustaceans (crabs, shrimps and prawns | Marais (1984) | |  |
| White seabream  (*Diplodus sargus* [DS]) | Primary Piscivore | Fishes, benthic crustaceans, molluscs, gastropods | Sala & Ballesteros (1997); Leitão et al. (2007) | |  |
| Bluefish  (*Pomatomus saltatrix* (PSalt) | Primary Piscivore | Fishes, crustaceans, cephalopods (squid and cuttlefish), mollusks | Marais (1984); Bennett (1989); Bowman &Center (2000); Lucena et al. (2000); Harding & Mann (2001) | |  |
| Bull ray  (*Pteromylaeus bovinus* [PB]) | Primary Piscivore | Fishes, cephalopods (squid and cuttlefish), crustaceans, bivalves, gastropods | Compagno et al. (1989) | |  |
| Greater guitarfish  (*Rhynchobatus* *djiddensis* [RD]) |  | Fishes, benthic crustaceans (shrimp, prawn, lobster) | Nasir (2000); Bornatowski et al. (2010) | |  |
| Speckled eagle ray  (*Aetobatus narinari* [AB]) | Primary piscivore | Gastropods, bivalves, crustaceans | Schluessel et al. (2010); Ajemian et al. (2012) | |  |
| **SECONDARY PISCIVORE** | | | | | |
| Milk  (*Rhizoprionodon acutus* [RA]) | Secondary piscivore | Elasmobranchs, fishes, molluscs, crustaceans | Gelsliechter et al. (1999); Hoffmayer & Parsons (2003); Bethea et al. (2004, 2006) | |  |
| Copper  (*Carcharhinus brachyurus* [CB]) | Secondary piscivore | Elasmobranchs, marine mammals (dolphin and whale), fishes, cephalopods, crustaceans | Smale (1991); Cliff & Dudley (1992); Lucifora et al. (2009) | |  |
| Smooth hammerhead  (*Sphyrna zygaena* [SZ]) | Secondary piscivore | Elasmobranchs, fishes, cephalopods (squids) | Smale (1991) | |  |
| Spinner  (*Carcharhinus brevipinna* [CBr]) | Secondary piscivore | Elasmobranchs, fishes, molluscs, cephalopods, crustaceans | Allen & Cliff (2000); Driggers et al. (2012) | |  |
| Dusky  (*Carcharhinus obscurus* [CO]) | Secondary piscivore | Elasmobranchs (large and small sharks and rays), marine mammals (dolphin and whale), fishes, cephalopods, crustaceans | Smale (1991); Gelsliechter et al. (1999); Dudley et al. (2005); Hussey at al. (2011) | |  |
| Scalloped hammerhead  (*Sphyrna lewini* [SL]) | Secondary piscivore | Elasmobranch, fishes, birds, cephalopods, crustaceans | deBruyn et al. (2005), Hussey et al. (2011) | |  |
| **TERTIARY PISCIVORE** | | | | | |
| White shark  (*Carcharodon carcharias* [CC]) | Tertiary piscivore | Elasmobranchs (large and small sharks and rays), marine mammals (seal, dolphin and whale), fishes, birds, cephalopods, crustaceans | Bass et al. (1975); Tricas & McCosker (1984); Klimley (1985); Cliff et al. (1989); Bruce (1992); Cliff et al. (1996); Hussey et al. (2012). | |  |
| Shortfin mako shark  (*Isurus oxyrinchus* [IO]) | Tertiary piscivore | Elasmobranchs (large and small shark and rays), fishes, cephalopods, crustaceans | Rogers et al. (2012); Wood et al. (2009); Maia et al. (2006); Cliff et al. (1990); | |  |
| Bull shark  (*Carcharhinus leucas* [CL]) | Tertiary piscivore | Elasmobranchs (large and small shark and rays), marine mammals (dolphins), fishes, birds, cephalopods, crustaceans | Snelson et al. (1984); Cliff & Dudley (1991a); Thorburn & Rowland (2008) | |  |
| Sand tiger shark  (*Carcharias taurus* [CT]) | Tertiary piscivore | Elasmobranchs (principally small sharks), fishes, cephalopods, crustaceans | Cliff (unpublished data), Gelsleichter et al. (1999); Smale (2005); Lucifora et al. (2009) | |  |
| Pigeye shark  (*Carcharhinus amboinensis* [CA]) | Tertiary piscivore | Elasmobranchs (large and small sharks and rays), marine mammals (dolphins), cephalopods and crustaceans | Cliff and Dudley (1991b) | |  |
| **CUMBERLAND SOUND, CANADIAN ARCTIC** | | | | | |
| **ZOOPLANKTIVORE** | | | | | |
| Capelin  (*Mallotus villosus* [MV]) | Zooplanktivore | Euphausiids, *Calanus* copepods | Vesin et al. (1981); Orlova et al. (2010) | |  |
| Herring  (*Clupea harengus* [CH]) | Zooplanktivore | Cladocerans, copepods | Dziaduch (2011) | |  |
| Shorthorn sculpin  (*Myoxocephalus scorpius* [MS]) | Invertivore (<24 cm) | Mysids, *Limacina helicina* | Cardinale (2000); McMeans (unpublished data) | |  |
| Arctic char  (*Salvelinus alpinus* [SA]) | Zooplanktivore (<50 cm) | *Parathemisto* zooplankton | Moore & Moore (1974) | |  |
| **PRIMARY PISCIVORE** |  |  |  | |  |
| Shorthorn sculpin  (*Myoxocephalus scorpius* [MS]) | Primary piscivore (>24 cm) | Fishes (Herring, larval sculpin) | Cardinale (2000); McMeans (unpublished data) | |  |
| Arctic char  (*Salvelinus alpinus* [SA]) | Primary piscivore (>50 cm) | Fishes (Arctic cod, capelin) | Moore & Moore (1974); McMeans (unpublished data) | |  |
| Arctic skate  (*Amblyraja hyperborea* [AH]) | Primary piscivore, | Fishes, cephalopods (squid-*Lebbeus polaris*) | Dolgov (2005); McMeans (unpublished data) | |  |
| Greenland halibut  (*Reinhardtius hippoglossoides* [RH]) | Primary Piscivore (<69 cm) | Fishes (capelin), Euphausiids, crustaceans, cephalopods | Dolgov (2002); Hovde et al. (2002); Solmundsson (2007) | |  |
| **SECONDARY PISCIVORE** | | | | | |
| Greenland halibut  (*Reinhardtius hippoglossoides* [RH]) | Secondary piscivore (>70 cm) | Fishes (eelpout, redfish, Arctic cod, Greenland halibut), scavenger (seal) | Rodríguez-Marín et al. (1995); Orr & Bowering (1997); Michalsen & Nedreaas (1998); Hovde et al. (2002); Solmundsson (2007); | |  |
| **TERTIARY PISCIVORE** |  |  |  | |  |
| Greenland shark  (*Somniosus microcephalus* [SM]) | Tertiary piscivore | Elasmobranchs (Greenland shark, Arctic skate), fishes (Greenland halibut, Arctic skate, sculpin, char, capelin), marine mammals (ringed seal, polar bear) | Fisk et al. (2002); Yano et al. (2007); McMeans et al. (2010); Leclerc et al. (2012) | |  |

**REFERENCES:**

Ajemian, M.J., Powers, S.P. & Murdoch, T.J.T. (2012). Estimating the potential impacts of large mesopredators on benthic resources: integrative assessment of spotted eagle ray foraging ecology in Bermuda. *PloS One*, 7, e40227. doi:10.1371/journal.pone.0040227.

Allen, B.R. & Clif, G. (2000). Sharks caught in the protective gill nets off Natal, South Africa. 9. The spinner shark *Carcharhinus brevipinna* (Müller and Henle). *S. Afr. J. Mar. Sci.*, 22, 199-215.

Bass, A.J., D'Aubrey, J.D. & Kistnasamy, N. (1975). Sharks of the east coast of Southern Africa. 4. the families Odontaspididae, Scapanorhynchidae, Isuridae, Cetorhinidae, Alopiidae, Orectolobidae and Rhiniodontidae. *Invest. Rep. Oceanogr. Res. Inst. Durban S. Afr.*, 39, 1-102.

Bennett, B. (1989). The diet of fish in three south-western Cape estuarine systems. *S. Afr. J. Zool.*, 24, 163-177.

Bethea, D.M., Buckel, J.A. & Carlson, J.K. (2004). Foraging ecology of the early life stages of four sympatric shark species. *Mar. Ecol. Prog. Ser.*, 268, 245-264.

Bethea, D.M., Carlson, J.K., Buckel, J.A. & Satterwhite, M. (2006). Ontogenetic and size-related trends in the diet of the Atlantic sharpnose shark *Rhizoprionodon terranovae* from the northeast Gulf of Mexico. *Bull. Mar. Sci.*, 78.

Bornatowski, H., de Castro Robert, M.C. & Costa, L. (2010). Feeding of guitarfish *Rhinobatos percellens* (Walbaum, 1972)(Elasmobranchii, Rhinobatidae), the target of artisanal fishery in Southern Brazil. *Braz. J. Oceanogr.*, 58, 45-52.

Bowman, R.E. & Center, N.F.S. (2000). *Food of northwest Atlantic fishes and two common species of squid*. National Oceanic and Atmospheric Administration, NMFS-NE 155, 138 p.

Brinton, E. & Townsend, A. (1980). Euphausiids in the Gulf of California-the 1957 cruises. *Calif. Coop. Oceanic Fish. Invest. Rep*, 21, 211-236.

Bruce, B.D. (1992). Preliminary observations on the biology of the white shark, Carcharodon carcharias, in south Australian waters. *Mar. Freshwater Res.*, 43, 1-11.

Cardinale, M. (2000). Ontogenetic diet shifts of bull-rout, *Myoxocephalus scorpius* (L.), in the south western Baltic Sea. *J. Appl. Ichtyol.*, 16, 231-239.

Clark, E. & Nelson, D.R. (1997). Young whale sharks, *Rhincodon typus*, feeding on a copepod bloom near La Paz, Mexico. *Environ. Biol. Fishes*, 50, 63-73.

Cliff, G., Dudley, S.F.J. & Davis, B. (1990). Sharks caught in the protective gill nets off Natal, South Africa. 3. The shortfin mako shark *Isurus oxyrinchus* (Rafinesque). *S. Afr. J. Mar. Sci.*, 9, 115-126.

Cliff, G. & Dudley, S. (1991a). Sharks caught in the protective gill nets off Natal, South Africa. 4. The bull shark *Carcharhinus leucas* Valenciennes. *S. Afr. J. Mar. Sci.*, 10, 253-270.

Cliff, G. & Dudley, S. (1991b). Sharks caught in the protective gill nets off Natal, South Africa. 5. The java shark *Carcharhinus leucas* (Müller and Henle). *S. Afr. J. Mar. Sci.*, 10, 253-270.

Cliff, G. & Dudley, S. (1992). Sharks caught in the protective gill nets off Natal, South Africa. 6. The copper shark *Carcharhinus brachyurus* (Günther). *S. Afr. J. Mar. Sci.*, 12, 663-674.

Cliff, G., Van Der Elst, R., Govender, A., Witthuhn, T. & Bullen, E. (1996). First estimates of mortality and population size of white sharks on the South African coast. In: *Great white sharks: the biology of Carcharodon carcharias* (eds. Klimley AP & D.G. A). Academic San Diego, pp. 393-400.

Compagno, L.J.V., Ebert, D.A. & Smale, M.J. (1989). *Guide to the sharks and rays of southern Africa*. New Holland, London, 158 pp.

de Bruyn, P., Dudley, S., Cliff, G. & Smale, M. (2005). Sharks caught in the protective gill nets off KwaZulu-Natal, South Africa. 11. The scalloped hammerhead shark *Sphyrna lewini* (Griffith and Smith). *Afr. J. Mar. Sci.*, 27, 517-528.

De Silva-Davila, R., Palomares-Garcia, R. & Hendrickx, M. (2002). Distributional patterns of the euphausiid community in Bahia de La Paz, BCS, Mexico. *Contributions to the study of the East Pacific crustaceans. Mexico: UNAM Instituto de Ciencias del Mar y LimnologiaMexico: UNAM Instituto de Ciencias del Mar y Limnologia*, 109-125.

Dolgov, A.V. (2002). The role of capelin (*Mallotus villosus*) in the food web of the Barents Sea. *ICES J. Mar. Sci.,* 59, 1034-1045.

Dolgov, A.V. (2005). Feeding and food consumption by the Barents Sea skates. *J. Northw. Atl. Fish. Sci.*, 35, 495-503.

Driggers, W.B., Campbell, M.D., Hoffmayer, E.R. and Ingram, G.W. (2012). Feeding chronology of six species of carcharhinid sharks in the western North Atlantic Ocean as inferred from longline capture data. *Mar. Ecol. Prog. Ser*., 465, 185-192.

Dudley, S., Cliff, G., Zungu, M. & Smale, M. (2005). Sharks caught in the protective gill nets off KwaZulu-Natal, South Africa. 10. The dusky shark *Carcharhinus obscurus* (Lesueur 1818). *Afr. J. Mar. Sci.*, 27, 107-127.

Duffy, C. (2002). Distribution, seasonality, lengths, and feeding behaviour of whale sharks (*Rhincodon typus*) observed in New Zealand waters. *N.Z. J. Mar. Freshwat. Res.*, 36, 565-570.

Dziaduch, D. (2011). Diet composition of herring (*Clupea harengus* L.) and cod (*Gadus morhua* L.) in the southern Baltic Sea in 2007 and 2008. *Oceanol. Hydrobiol.*, 40, 96-109.

Espinoza, P., Bertrand, A., Van Der Lingen, C.D., Garrido, S. & Rojas de Mendiola, B. (2009). Diet of sardine (*Sardinops sagax*) in the northern Humboldt Current system and comparison with the diets of clupeoids in this and other eastern boundary upwelling systems. *Prog. Oceanogr.*, 83, 242-250.

Fischer, W., Sousa, I., Silva, A., de Freitas, A., Poutiers, J.M., Schneider, W., Borges, T.C., Feral, J.P. & Massinga, A. (1990). Fichas FAO de identificaçao de espécies para actividades de pesca. Guia de campo das espécies comerciais marinhas e de águas salobras de Moçambique. Publicaçao preparada em collaboraçao com o Instituto de Investigaçao Pesquiera de Moçambique, com financiamento do Projecto PNUD/FAO MOZ/86/030 e de NORAD. Roma, FAO. 1990. 424 p.

Fisk, A.T., Tittlemier, S.A., Pranschke, J.L. & Norstrom, R.J. (2002) Using anthropogenic contaminants and stable isotopes to assess the feeding ecology of Greenland sharks. *Ecology*, 83, 2162-2172.

Gelsleichter, J., Musick, J.A. & Nichols, S. (1999). Food habits of the smooth dogfish, *Mustelus canis*, dusky shark, *Carcharhinus obscurus*, Atlantic sharpnose shark, *Rhizoprionodon terraenovae*, and the sand tiger, *Carcharias taurus*, from the northwest Atlantic Ocean. *Environ. Biol. Fishes*, 54, 205-217.

Graham, B.S., Grubbs, D., Holland, K. & Popp, B.N. (2007). A rapid ontogenetic shift in the diet of juvenile yellowfin tuna from Hawaii. *Mar. Biol.*, 150, 647-658.

Griffiths, M. (1997). Feeding ecology of South African *Argyrosomus japonicus* (Pisces: Sciaenidae), with emphasis on the Eastern Cape surf zone. *S. Afr. J. Mar. Sci.*, 18, 249-264.

Hacohen-Domene, A., Galvan-Magana, F. & Ketchum-Mejia, J. (2006). Abundance of whale shark (*Rhincodon typus*) preferred prey species in the southern Gulf of California, Mexico. *Cybium*, 30, 99-102.

Harding, J.M. & Mann, R. (2001). Diet and habitat use by bluefish, *Pomatomus saltatrix*, in a Chesapeake Bay estuary. *Environ. Biol. Fishes*, 60, 401-409.

Havelange, S., Lepoint, G., Dauby, P. & Bouquegneau, J.M. (1997). Feeding of the sparid fish *Sarpa salpa* in a seagrass ecosystem: diet and carbon flux. *Mar. Ecol.*, 18, 289-297.

Heyman, W.D., Graham, R.T., Kjerfve, B. & Johannes, R.E. (2001). Whale sharks *Rhincodon typus* aggregate to feed on fish spawn in Belize. *Mar. Ecol. Prog. Ser.*, 215, 275-282.

Hoffmayer, E.R. & Parsons, G.R. (2003). Food habits of three shark species from the Mississippi Sound in the northern Gulf of Mexico. *Southeast. Nat.*, 2, 271-280.

Hovde, S.C., Albert, O.T. & Nilssen, E.M. (2002). Spatial, seasonal and ontogenetic variation in diet of Northeast Arctic Greenland halibut (*Reinhardtius hippoglossoides*). *ICES J. Mar. Sci.,* 59, 421-437.

Hussey, N.E., Dudley, S.F.J., McCarthy, I.D., Cliff, G. & Fisk, A.T. (2011). Stable isotope profiles of large marine predators: viable indicators of trophic position, diet, and movement in sharks? *Can. J. Fish. Aquat.Sci.*, 68, 2029-2045.

Hussey, N.E., McCann, H.M., Cliff ,G., Dudley, S.F.J., Wintner, S.P. & Fisk, A.T. (2012). Size-Based Analysis of Diet and trophic Position of the White Shark, *Carcharodon carcharias*, in South African Waters. In: *Global Perspectives on the Biology and Life History of the White Shark* (ed. Domeier M). CRC Boca Rotan, pp. 27-49.

Jarman, S. & Wilson, S. (2004). DNA-based species identification of krill consumed by whale sharks. *J. Fish Biol.*, 65, 586-591.

Klimley, A.P. (1985). The areal distribution and autecology of the white shark, *Carcharodon carcharias,* off the West Coast of North America. *Mem. South Calif. Acad. Sci.*, 9, 15-40.

Kuhnert, P.M., Duffy, L.M., Young, J.W. & Olson, R.J. (2012). Predicting fish diet composition using a bagged classification tree approach: a case study using yellowfin tuna (*Thunnus albacares*). *Mar. Biol.*, 159, 87-100.

Lechanteur, Y. & Griffiths, C. (2003). Diets of common suprabenthic reef fish in False Bay, South Africa. *Afr. Zool.*, 38, 213-227.

Leclerc, M.E., Lydersen, C, Haug, T., Bacnmann, L., Fisk, A.T. & Kovacs, K.M. (2012). A missing piece in the Arctic food web puzzle? Stomach contents of Greenalnd sharks sampled in Svalbard, Norway. Pol. Biol., 35, 1197-1208.

Leitão, F., Santos, M.N. & Monteiro, C.C. (2007). Contribution of artificial reefs to the diet of the white sea bream (*Diplodus sargus*). *ICES J. Mar. Sci.*, 64, 473-478.

Lucena, F.M., Vaske, T., Ellis, J.R. & O'Brien, C.M. (2000). Seasonal variation in the diets of bluefish, *Pomatomus saltatrix* (Pomatomidae) and striped weakfish, *Cynoscion guatucupa* (Sciaenidae) in southern Brazil: implications of food partitioning. *Environ. Biol. Fishes*, 57, 423-434.

Lucifora L.O., Garcia V.B., Menni R.C., Escalante A.H. & Hozbor N.M. (2009). Effects of body size, age and maturity stage on diet in a large shark: ecological and applied implications. *Ecol. Res.*, 24, 109-118.

Lucifora, L.O., Garcia, V.B. & Escalante, A.H. (2009). How can the feeding habits of the sand tiger shark influence the success of conservation programs? Anim. Consrv., 12, 291-301.

Maia, A., Queiroz, N., Correia, J.P. & Cabral, H. (2006). Food habits of the shortfin mako, *Isurus oxyrinchus*, off the southwest coast of Portugal. *Env. Biol. Fish.,* 77, 157-167.

Marais, J. (1984). Feeding ecology of major carnivorous fish from four eastern Cape estuaries. *S. Afr. J. Zool.*, 19, 210-223.

McMeans, B.C., Svavarsson, J., Dennard, S.T. & Fisk, A.T. (2010). Diet and resource use among Greenland sharks (*Somniosus microcephalus*) and teleosts sampled in Icelandic waters, using δ^13^C, δ^15^N and total mercury. *Can. J. Fish. Aquat. Sci.,* 67, 1428-1438.

Meekan, M., Jarman, S., McLean, C. & Schultz, M. (2009). DNA evidence of whale sharks (*Rhincodon typus*) feeding on red crab (*Gecarcoidea natalis*) larvae at Christmas Island, Australia. *Mar. Freshwater Res.*, 60, 607-609.

Michalsen, K. & Nedreaas, K.H. (1998). Food and feeding of Greenland halibut (*Reinhardtius hippoglossoides*, Walbaum) in the Barents Sea and East Greenland waters. *Sarsia*, 83, 401-407.

Moore, J. & Moore, I. (1974). Food and growth of Arctic char, *Salvelinus alpinus* (L.), in the Cumberland Sound area of Baffin Island. *J. Fish Biol.*, 6, 79-92.

Motta, P.J., Maslanka, M., Hueter, R.E., Davis, R.L., De La Parra, R., Mulvany, S.L., Habegger, M.L., Strother, J.A., Mara, K.R. & Gardiner, J.M. (2010). Feeding anatomy, filter-feeding rate, and diet of whale sharks *Rhincodon typus* during surface ram filter feeding off the Yucatan Peninsula, Mexico. *Zoology*, 113, 199-212.

Nasir, N.A. (2000). The food and feeding relationships of the fish communities in the inshore waters of Khor Al-Zubair, northwest Arabian Gulf. *Cybium*, 24, 89-99.

Notarbartolo-di-Sciara, G. (1988). Natural history of the rays of the genus *Mobula* in the Gulf of California. *Fish. Bull.*, 86, 45-66.

Orlova, E.L., Rudneva, G.B., Renaud, P.E., Eiane, K., Savinov, V. & Yurko, A.S. (2010). Climate impacts on feeding and condition of capelin *Mallotus villosus* in the Barents Sea: evidence and mechanisms from a 30 year data set. *Aquat. Biol.*, 10, 105-118.

Orr, D. & Bowering, W. (1997). A multivariate analysis of food and feeding trends among Greenland halibut (*Reinhardtius hippoglossoides*) sampled in Davis Strait, during 1986. *ICES J. Mar. Sci.*, 54, 819-829.

Rodríguez-Marín, E., Punzón, A.& Paz, J. (1995). Feeding patterns of Greenland halibut (*Reinhardtius hippoglossoides*) in Flemish Pass (Northwest Atlantic). *NAFCO Sci. Coun. Studies*, 23, 43-54.

Rogers, P.J., Huveneers, C., Page, B., Hamer, D.J., Goldsworthy, S.D., Mitchell, J.G & Seuront, L. (2012). A quantitative comparison of the diets of sympatric prlagic sharks in the gulf and shelf ecosystems off southern Australia. *ICES J. Mar. Sci.*, 69, 1382-1393.

Rohit, P., Rao, G.S. & Rammohan, K. (2011). Feeding strategies and diet composition of yellowfin tuna *Thunnus albacares* (Bonnaterre, 1788) caught along Andhra Pradesh, east coast of India. *Indian J. Fish.*, 57, 13-20.

Rudershausen, P.J., Buckel, J.A., Edwards, J., Gannon, D.P., Butler, C.M. & Averett, T.W. (2010). Feeding ecology of blue marlins, dolphinfish, yellowfin tuna, and wahoos from the North Atlantic Ocean and comparisons with other oceans. *Trans. Am. Fish. Soc.*, 139, 1335-1359.

Sala, E. & Ballesteros, E. (1997). Partitioning of space and food resources by three fish of the genus *Diplodus* (Sparidae) in a Mediterranean rocky infralittoral ecosystem. *Mar. Ecol. Prog. Ser.*, 152, 273-283.

Schluessel, V, Bennett, M.B. & Colin, S.P. (2010). Diet and reproduction in the white-spotted eagle ray Aetobatus narinari from Queensland, Australia and the Penghu Isalnds, Taiwan. *Mar. Freshwater Res.*, 61, 1278-1289.

Silas, E. & Rajagopalan, M. (1963). On a recent capture of a whale shark (*Rhincodon typus* Smith) at Tuticorin, with a note on information to be obtained on whale sharks from Indian waters. *J. Mar. Biol. Ass. India*, 5, 153-157.

Smale, M.J. (1986). The feeding habits of six pelagic and predatory teleosts in eastern Cape coastal waters (South Africa). *J. Zool.*, 1, 357-409.

Smale, M. (1991). Occurrence and feeding of three shark species, *Carcharhinus brachyurus*, *C. obscurus* and *Sphyrna zygaena*, on the Eastern Cape coast of South Africa. *S. Afr. J. Mar. Sci.*, 11, 31-42.

Smale, M. (2005). The diet of the ragged-tooth shark *Carcharias taurus* Rafinesque1810 in the Eatsern Cape, South Africa. *Afr. J. Mar. Sci.*, 27, 331-335

Snelson, F.F., Mulligan, T.J. & Williams, S.E. (1984). Food Habits, Occurrence, and Population Structure of the Bull Shark, *Carcharhinus Leucas*, in Florida Coastal Lagoons. *Bull. Mar. Sci.*, 34, 71-80.

Solmundsson, J. (2007). Trophic ecology of Greenland halibut (*Reinhardtius hippoglossoides*) on the Icelandic continental shelf and slope. *Mar. Biol. Res.*, 3, 231-242.

Taylor, G. (1994). *Whale sharks: the giants of Ningaloo Reef*. Angus & Robertson, Sydney.

Taylor, J. (1996). Seasonal occurrence, distribution and movements of the whale shark, *Rhincodon typus*, at Ningaloo Reef, Western Australia. *Mar. Freshwater Res.*, 47, 637-642.

Taylor, J. & Pearce, A. (1999). Ningaloo Reef currents: implications for coral spawn dispersal, zooplankton and whale shark abundance. *J. R. Soc. West. Aust.*, 82, 57-65.

Taylor, J.G. (2007). Ram filter-feeding and nocturnal feeding of whale sharks (*Rhincodon typus*) at Ningaloo Reef, Western Australia. *Fish. Res.*, 84, 65-70.

Thorburn, D.C. & Rowland, A.J. (2008). Juvenile bull sharks 'Carcharhinus leucas' (Valenciennes, 1839) in northern Australian rivers. *The Beagle: Records of the Museums and Art Galleries of the Northern Territory*, 24, 79-86.

Tricas, T.C. & Maccosker, J.E. (1984). Predatory behavior of the white shark (*Carcharodon carcharias*) with notes on its biology. *Proc. Calif. Acad. of Sci.,* 43, 221-238.

Van der Elst, R. (1986). Guide to common sea fishes of Southern Africa. C. Struik, Cape Town, pp. 386.

Van der Elst, R. & Adkin, F. (1991). *Marine linefish: priority species and research objectives in southern Africa*. Oceanogr. Res. Inst., Spec. Publ. No.1, 132pp.

Van der Lingen, C. (2002). Diet of sardine *Sardinops sagax* in the southern Benguela upwelling ecosystem. *S. Afr. J. Mar. Sci.*, 24, 301-316.

Van Der Lingen, C., Coetzee, J. & Hutchings, L. (2010). Overview of the KwaZulu-Natal sardine run. *Afr. J. Mar. Sci.*, 32, 271-277.

Van Der Westhuizen, H.C. & Marais, J.F.K. (1977). Stomach content analysis of *Pomadasys commersonnii* from the Swartkops Estuary (Pisces: Pomadasidae). *Zool. Afr.*, 12, 500-504.

Vesin, J.P., Leggett, W.C. & Able, K.W. (1981). Feeding ecology of capelin (*Mallotus villosus*) in the Esturary and Western Gulf of St. Lawrence and its multispecies interactions. *Can. J. Fish. Aquat. Sci*., 38, 257-267.

Wilson, S.G. & Newbound, D.R. (2001). Two whale shark faecal samples from Ningaloo Reef, Western Australia. *Bull. Mar. Sci.*, 68, 361-362.

Wood, A.D., Wetherbee, B., Juanes, F., Kohler, N.E. & Wilga, C. (2009) Recalculated diet and daily ration of the shortfin mako (Isurus oxyrinchus), with a focus on quantifying predation on blusfish (Pomatomus saltatrix) in the northwest Atlantic Ocean. *Fish. Bull.*, 107, 76-88.

Yano, K., Stevens, J.D. & Compagno, L.J.V. (2007). Distribution, reproduction and feeding of the Greenland shark *Somniosus (Somniosus) microcephalus*, with notes on two other sleeper sharks, *Somniosus (Somniosus) Pacificus* and *Somniosus (Somniosus) antarcticus*. *J. Fish. Biol*., 70, 374-390.
