# Supplementary material for: Rescaling the trophic structure of marine food webs
Source: Ecol Lett. 2013 Dec 6;17(2):239–50. doi: 10.1111/ele.12226 (PMC3912912; doi:10.1111/ele.12226)
Supplement: Supplementary file 7 [file ele0017-0239-sd7.docx]

**Supplementary Material S7**

*Mechanism driving a scaled Δ^15^N value*

A well-studied analogy to isotopic incorporation is the accumulation of hydrophobic contaminants, such as PCBs, through food webs. The increase in concentration of a PCB from food to a consumer, called a biomagnification factor (BMF) is a non-additive value, the mechanisms of which are well characterized (Kelly *et al.* 2004). Consequently, PCBs do not increase consistently with each trophic step but instead relate to the concentration of the TL one step below. This is a result of a summation of uptake and elimination rates and source pool concentrations, which can be variable (Fisk *et al.* 1998). As the processes associated with nitrogen stable isotope incorporation into an animal’s tissues are dependent on rates of assimilation and elimination, the source pool of δ^15^N for an organism is likely to affect subsequent discrimination through the food web. Few studies have quantified both uptake and elimination rates for ^15^N/^14^N and ^13^C/^12^C, but such data could be key to a mechanistic understanding of stable isotope dynamics (see Supplementary Materials S3).

**REFERENCES:**

Kelly, B.C., Gobas, F.A.P.C. & McLachlan, M.S., (2004). Intestinal absorption and biomagnification of organic contaminants in fish, wildlife, and humans. *Environ. Toxicol. Chem.*, 23, 2324-2336.

Fisk, A.T., Norstrom, R.J., Cymbalisty, C.D., & Muir, D.C.G. (1998). Dietary accumulation and depuration of hydrophobic organochlorines: Bioaccumulation parameters and their relationship with the octanol/water partition coefficient. *Environ. Toxicol. Chem.*, 17, 951-961.
